# Targeting sphingolipid metabolism: inhibition of neutral sphingomyelinase 2 impairs coronaviral replication organelle formation

**DOI:** 10.1128/mbio.00084-25

**Published:** 2025-08-14

**Authors:** Florian Salisch, Fabian Schumacher, Ulrich Gärtner, Burkhard Kleuser, John Ziebuhr, Christin Müller-Ruttloff

**Affiliations:** 1 Institute of Medical Virology, Justus Liebig University Giessen 9175, Giessen, Germany; 2 Institute of Pharmacy, Freie Universität Berlin 9166, Berlin, Germany; 3 Institute of Anatomy and Cell Biology, Justus Liebig University Giessen 9175, Giessen, Germany; Charite-Universitatsmedizin Berlin, Berlin, Germany

**Keywords:** coronavirus, replication organelles, viral replication, sphingomyelinase, lipid metabolism, HCoV-229E

## Abstract

Many plus-strand RNA viruses, including coronaviruses, orchestrate the formation of specialized replication organelles in the cytoplasm of infected cells by specific membrane-associated viral nonstructural proteins (nsp’s). This process involves a massive reorganization of intracellular membrane compartments and is thought to depend on a specific set of cellular enzymes that synthesize or modify cellular lipids. Here, we investigated the potential role(s) of specific sphingolipids and related enzymes in coronavirus replication. We observed in cells infected with three different coronaviruses a comparable increase in global ceramide levels, whereas sphingomyelin levels were reduced, suggesting an enhanced conversion of sphingomyelin to ceramide by sphingomyelinases. To test this hypothesis, we targeted cellular sphingomyelinases using pharmacological and genetic approaches. The data obtained in these experiments led us to conclude that neutral sphingomyelinase 2 plays a critical role in an early (but not the entry) step of coronavirus replication in Huh-7-ACE2 cells. Furthermore, neutral sphingomyelinase 2 inhibited the formation of viral replication organelles, indicating the involvement of ceramide in the formation of these membrane structures. We also confirmed colocalization of neutral sphingomyelinase 2 and ceramide, but not sphingomyelin, with replication organelles that were produced in coronavirus-infected cells or induced by co-expression of coronavirus nsp3 and nsp4. A colocalization of ceramides with coronaviral replication organelles could be consistently demonstrated for different epithelial cell systems using genetically diverse coronaviruses, describing potential cell- and virus-type independence.

**IMPORTANCE:**

Coronaviruses are enveloped plus-strand RNA viruses with a broad host range, including humans. Human coronaviruses are not only associated with endemic, mild upper respiratory tract infections but also have pandemic potential and can be associated with a severe disease burden. The recent SARS-CoV-2 pandemic especially highlighted the urgent need to identify ideal broad-spectrum and host-targeted antiviral strategies. Since lipids are involved in every step of viral replication, we compared changes in the sphingolipid metabolism of cells infected with three different coronaviruses to identify similarities and related cellular enzymes involved in facilitating viral replication. We observed increased ceramide levels while sphingomyelin levels decreased, suggesting enhanced sphingomyelin-to-ceramide conversion by cellular sphingomyelinases upon infection. Impairment of neutral sphingomyelinase 2 reduced viral replication and the formation of virus-induced membranous replication organelles. Furthermore, we found that neutral sphingomyelinase 2 and its product ceramide were associated with viral replication organelles. Ceramides consistently appeared to be integral lipid building blocks of replication organelles across different human pathogenic coronaviruses and cell types. In conclusion, our study provides new insights into novel potentially conserved druggable sphingolipid-related host factors involved in coronaviral replication, offering potential new targets for antiviral therapies against newly emerging coronaviruses.

## INTRODUCTION

Coronaviruses (CoVs) are enveloped plus-strand RNA (+ssRNA) viruses with a broad host range, including humans (1). The four endemic human CoVs (HCoV-229E, -NL63, -HKU1, and -OC43) generally cause a seasonal mild infection mainly restricted to the upper respiratory tract (2–5). In contrast, the three highly pathogenic CoVs that have emerged in the last two decades, including severe acute respiratory syndrome CoV (SARS-CoV) (6, 7), SARS-CoV-2 (8, 9), and Middle East respiratory syndrome CoV (MERS-CoV) (10), have often been associated with significant disease burden and mortality in humans.

The recent SARS-CoV-2 pandemic highlights the urgent need to identify new broad-spectrum (including host-targeted) antiviral strategies (11). Since CoVs interfere with a wide range of cellular pathways, there is increasing evidence that some of these pathways may be exploited therapeutically (12).

In common with all viruses, CoVs utilize the lipid metabolism of infected host cells (13, 14). Each replication step is closely associated with cellular membranes, including entry, fusion, replication, protein translation, assembly, and budding (15). Therefore, it is not surprising that viruses dynamically interfere with the heterogeneous composition of host cell membranes to fuel their replication. +ssRNA viruses especially induce extensive host membrane rearrangements, leading to the formation of membranous microenvironments in the cytoplasm of infected cells. Inside these microenvironments, viral replication and transcription take place (14). Indeed, these so-called replication organelles (ROs) are thought to provide a structural scaffold for the viral RNA synthesis machinery and contribute to sequestering viral components from host defense mechanisms, suggesting essential roles for ROs in viral replication (16, 17). Previous electron tomography studies revealed that CoVs induce mainly double-membrane vesicles (DMVs) and convoluted membranes that are interconnected with each other and the endoplasmic reticulum (ER), leading to the formation of large reticulovesicular membrane networks in infected cells (16, 18–20).

As the name suggests, DMVs are small vesicles of about 100–300 nm in diameter, surrounded by two membranes. In these membranes, molecular pores are embedded to provide, most likely, a transport route for viral RNA to exit the RO for translation and packaging (21, 22). Interestingly, the membrane-remodeling steps (required for RO formation) are mainly triggered by two viral nonstructural proteins (nsp’s), nsp3 and nsp4 (23). Nsp3 is a multifunctional protein with several functions (including a papain-like protease domain) and was recently identified as a major constituent of the RO pore complex (21, 22). In addition to nsp3, its interaction partner nsp4 is also known to induce membrane pairing and pore complex formation and is involved in anchoring the viral replication/transcription complex to cellular membranes (24). Both proteins contain conserved transmembrane domains and can induce (upon ectopic co-expression and in conjunction with host proteins/lipids) DMV-like ROs (23, 25, 26). Overall, the formation of CoV-induced membranous structures is still poorly understood, and the specific roles of membrane lipids and associated cellular factors remain largely unknown.

For hepaci- and flaviviruses, which induce comparable ER-derived ROs, sphingolipids are proposed to contribute to their formation (27–29). Sphingolipids are, besides glycerophospholipids, major structural components of biological membranes whose chemical features modulate the physical properties of lipid bilayers and control many membrane-associated cellular processes, including intracellular trafficking, vesicle formation, and signal transduction by membrane receptors (30, 31). Moreover, various sphingolipid species can be deregulated upon cellular stress conditions or in response to multiple stimuli, including viral infections (32–34).

To shed light on the potential involvement of (sphingo)lipids in CoV infection and systemically map host lipid-virus interaction networks in coronavirus-infected cells, we performed a sphingolipid analysis of Huh-7-ACE2 cells infected with three different human pathogenic CoVs (HCoV-229E, MERS-CoV, and SARS-CoV-2). We could demonstrate that CoV infection leads to a similar pattern of significant and time-dependent deregulations of cellular sphingolipid composition. Notably, upon CoV infection, total ceramide (Cer) levels drastically increased while simultaneously sphingomyelin (SM) species decreased, indicating the involvement of sphingomyelinases (SMases), the main driver of SM to Cer conversion. Furthermore, we identified a significant role of neutral sphingomyelinase 2 (nSMase2) in the replication of all three CoVs in Huh-7-ACE2 cells. Most likely, nSMase2 contributes to viral RO formation since (i) nSMase2 inhibition reduced RO formation and (ii) nSMase2 and the enzymatic product Cer (but not the substrate SM) colocalized with infection-induced or artificially induced ROs. Notably, Cer also colocalized with coronaviral ROs in lung-derived permanent and primary cell cultures. In addition, Cer upregulation, albeit to a lesser extent, was also observed in lung-derived cells following CoV infection.

Taken together, our study identified sphingolipid deregulations with a novel proviral role in CoV replication. We demonstrated that Cer species, produced or recruited via nSMase2-dependent or -independent pathways, serve as structural components in the formation of coronaviral ROs. Based on these data, it seems reasonable to suggest that targeting the sphingolipid metabolism may provide a possible therapeutic strategy to improve the progression and clinical outcome of coronavirus infections.

## RESULTS

### CoVs alter the sphingolipid homeostasis of infected host cells

**Fig 1:**
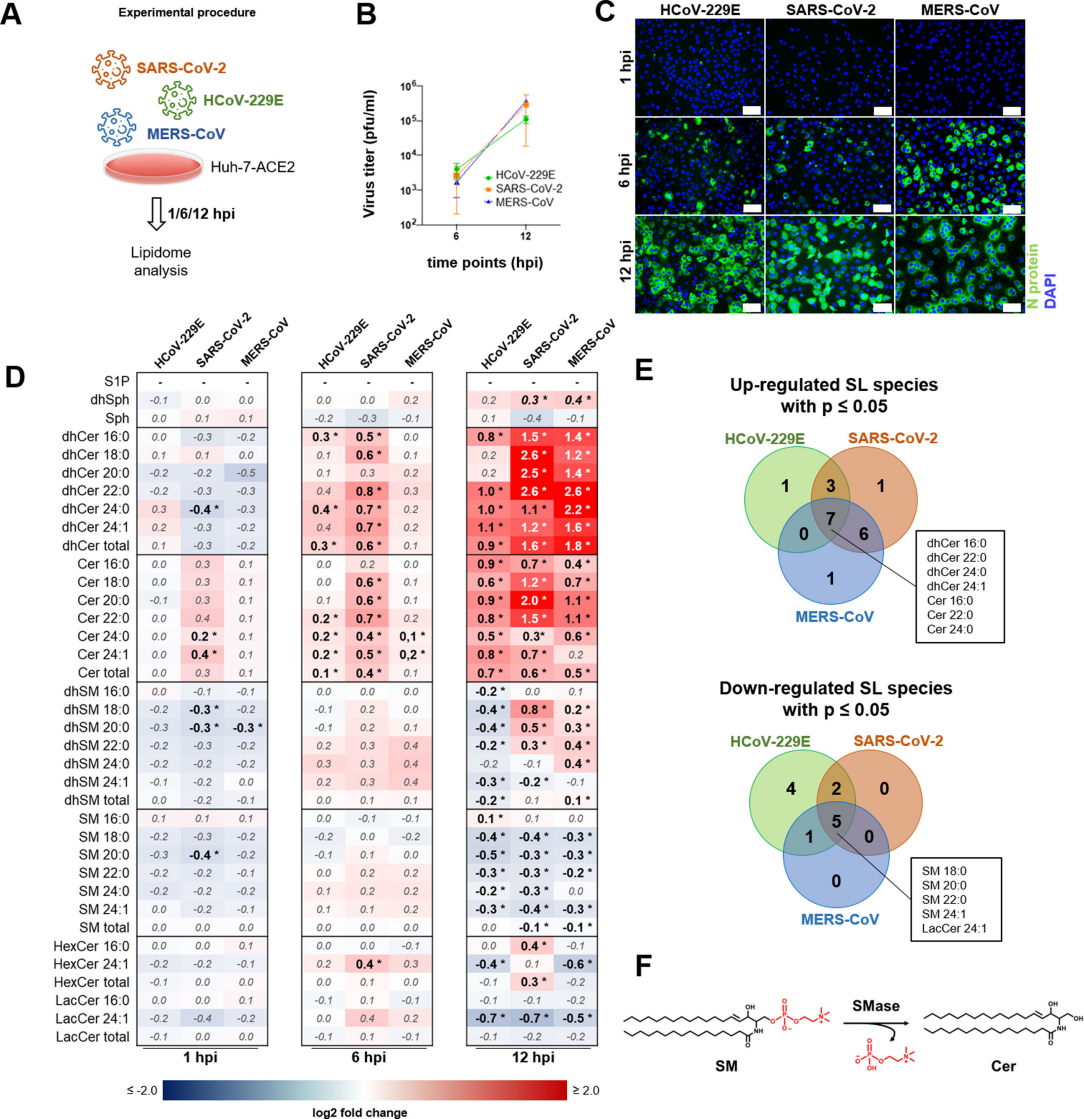
Time-dependent global cellular sphingolipid (SL) changes upon infection with three different CoVs. (**A**) Experimental design of the sphingolipidome analysis. Huh-7-ACE2 cells were mock infected or infected with the indicated CoV (multiplicity of infection [MOI] = 3) for 1, 6, and 12 hpi. (**B and C**) Corresponding growth kinetics and immunofluorescence images. Scale bars = 100 µm. (**D**) Heat maps showing fold changes of deregulated SL species at the indicated time points in relation to uninfected control (significant differences [*P* ≤ 0.05] in bold and marked with asterisks) calculated from the replicates by one-way analysis of variance (ANOVA) with Dunnett´s test for multiple comparisons. (**E**) Corresponding Venn diagrams. Experiments were done in quintuplicates (*n* = 5). (**F**) Simplified illustration of SL metabolism. Ceramide (Cer), as the centerpiece of the SL metabolic pathway, can be synthesized *de novo* via dhCer, via salvage pathway through hydrolysis of glycosphingolipids or by the sphingomyelinase (SMases) pathway through the hydrolysis of SM. Cer, ceramide; dhCer, dihydroceramide; dhSM, dihydrosphingomyelin; dhSph, dihydrosphingosine; HexCer, hexosylceramide; LacCer, lactosylceramide; S1P, sphingosine-1-phosphate; SM, sphingomyelin; Sph, sphingosine.

Since lipids are involved in nearly every step of the viral life cycle, we aimed to better understand lipid metabolism in CoV-infected cells and, in this study, decided to focus on cellular sphingolipid composition. For this, we performed a time-dependent sphingolipidomic study and determined sphingolipid changes in infected Huh-7 hepatoma cells overexpressing the human ACE2 receptor (Huh-7-ACE2) at three different time points post-infection (pi) using uninfected cells as control (Fig. 1A). We decided to use Huh-7 cells for comparative reasons because the three human alpha- (HCoV-229E) and betacoronaviruses (MERS-CoV and SARS-CoV-2) used in this study replicate with similar replication kinetics (Fig. 1B) and infection rates (>75%; Fig. 1C) in these cells.

We quantified 31 sphingolipid species, of which only a few species were significantly deregulated 1 hpi. At 6 hpi, 5 (for HCoV-229E), 11 (for SARS-CoV-2), and 2 (for MERS-CoV) sphingolipid species showed significant changes in their abundance. At 12 hpi, more than ~70% of all analyzed sphingolipid species were significantly altered (Fig. 1D), suggesting a time-dependent deregulation of cellular sphingolipid homeostasis in infected cells. A similar deregulation pattern was observed for all three CoVs. More specifically, we identified a global downregulation of SMs, whereas cellular Cer species were increased in infected cells (Fig. 1D and E). In all three CoVs, three Cer species (including the most common Cer species 16:0) and four dihydroceramide (dhCer) species were upregulated. In addition, four SM species were simultaneously found to be downregulated (Fig. 1E), indicating a conserved pattern of sphingolipid dysregulation in CoV infection.

The cellular enzymatic class of SMases drives the conversion of SM to Cer and phosphorylcholine (Fig. 1F). There are three major classes of SMases: acid, neutral, and alkaline SMase, which were classified according to the pH optimum of their activity. Since alkaline SMase is found exclusively in the intestinal tract and bile, we excluded it from our study and focused on acid SMase (aSMase) and the three major forms of neutral SMase (nSMase).

### Inhibition of nSMases impairs the replication of three different CoVs in Huh-7-ACE2 cells

**Fig 2:**
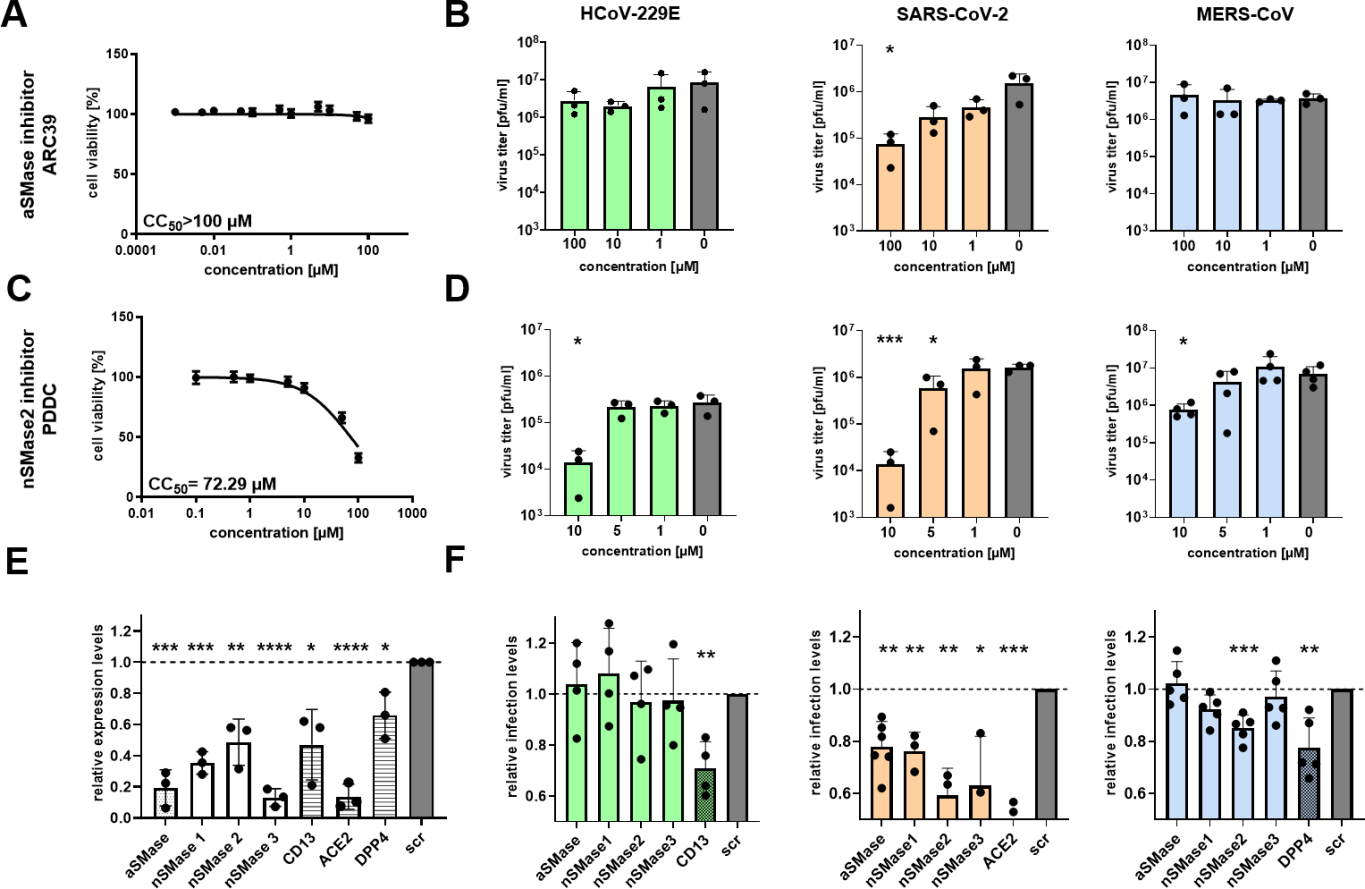
Antiviral activities of a/nSMase inhibition in CoVs replication. (**A through D**) Huh-7-ACE2 cells were mock-infected (**A and C**) or infected with the indicated virus (MOI = 0.1; **B and D**) in the presence of increasing concentrations of SMase inhibitors ([**A and B**] ARC39 for aSMase and [**C and D**] PDDC for nSMase2) or dimethyl sulfoxide (DMSO) as solvent control. Cell viability (**A and C**) or virus titers (**B and D**) in the presence of increasing inhibitor concentrations were determined using 3-(4,5-dimethyl-2-thiazolyl)-2,5-diphenyl-2H-tetrazolium bromide assay or plaque assay. (**E and F**) Genetic manipulation of SMases or CoV-specific entry receptors using siRNA knockdown. (**E**) Huh-7-ACE2 cells were transfected with the indicated siRNAs, and the target mRNA was analyzed using qPCR. (**F**) Impact of siRNA silencing on viral replication. Huh-7-ACE2 cells were reverse transfected with siRNAs (100 nM) for 48 h before being infected with the indicated virus. Infectivity was assessed by image-based quantification of N-positive cells and was normalized to levels in cells targeted by scrambled (scr) siRNA controls. All experiments were performed in Huh-7-ACE2 cells mock-infected or infected with the indicated virus at an MOI of 0.1 in three independent replicates. All bar graphs show mean ± SD; asterisks indicate *P* values (*P < 0.05; **P < 0.005; ***P < 0.0005) obtained by a two-tailed unpaired *t*-test.

To determine the functional consequences of the observed sphingolipid deregulation, we next investigated the effects of aSMase and nSMase inhibition on coronaviral replication using specific pharmacological inhibitors and a small-interfering RNA (siRNA)-based gene silencing approach (Fig. 2).

First, we used the specific aSMase inhibitor ARC39 (35), which showed no cytotoxicity up to 100 µM for 24 h in Huh-7-ACE2 cells (Fig. 2A). Interestingly, aSMase inhibition impaired the replication of SARS-CoV-2 (Fig. 2B, orange bars), which was consistent with the SARS-CoV-2 data reported previously (36, 37) but did not significantly affect HCoV-229E or MERS-CoV replication (Fig. 2B, green and blue bars). This conclusion was further supported using a second aSMase-specific inhibitor (PCK310 [38]), which impaired the replication of SARS-CoV-2 but not that of the other CoVs used in this experiment (Fig. S1A and B).

Next, we assessed the effect of nSMase inhibition by using pharmacological inhibitors. Two broadly active nSMase inhibitors, 3-O-methyl-sphingomyelin (39, 40) and GW4869 (41, 42), were revealed to reduce dose dependently and at non-toxic concentrations the replication of all three CoVs (Fig. S1C through F). Because nSMase2 is considered the major and best-characterized SM-hydrolyzing enzyme (43), we focused on this particular enzyme and used the highly specific nSMase2 inhibitor PDDC (44). Consistent with the data reported above for broadly active nSMase inhibitors, the nSMase2-specific inhibitor impaired the replication of all three CoVs at non-toxic concentrations (Fig. 2C and D), suggesting a coronavirus-wide conserved role for nSMase2 in coronaviral replication in Huh-7-ACE2 cells.

Although the inhibitors used in these experiments have well-documented substrate specificities, we used an additional siRNA-based gene silencing approach to corroborate the SMase inhibition data. For this, we targeted the transcripts of the aSMase and three nSMases. As controls, we used siRNA pools targeting the transcripts of the major cellular receptor of the three CoVs used in these experiments (CD13, also known as APN, for HCoV-229E [45]; DPP4 for MERS-CoV [46], and ACE2 for SARS-CoV-2 [47]). First, we analyzed (at 48 h post-transfection) the knockdown efficiencies of the respective siRNA pools used for the different mRNA targets. As shown in Fig. 2E, all siRNA pools reduced the corresponding mRNA levels significantly but to a varying extent.

At 48 h post-transfection of the siRNA pools, the cells were infected with the respective CoV, and infection rates were determined by automated imaging. Interestingly, HCoV-229E replication remained unaffected in the presence of siRNAs targeting cellular SMase transcripts (Fig. 2F, green bars). A possible compensatory effect of other SMases could explain this. In contrast, all SMase-targeting siRNAs used in this experiment were found to reduce SARS-CoV-2 infectivity, with nSMase2 knockdown displaying the most significant impact on viral infectivity (Fig. 2F, orange bars), confirming the pharmacological inhibitor data. For MERS-CoV, only the nSMase2 knockdown was found to impair MERS-CoV infection (Fig. 2F, blue bars), supporting the nSMase inhibitor data. Taken together, the data lead us to conclude that cellular nSMase(s), especially nSMase2, seem to promote CoV replication, albeit to a slightly varying extent.

### nSMase2 activity supports an early step of viral replication and may be involved in the formation of replication organelles

**Fig 3:**
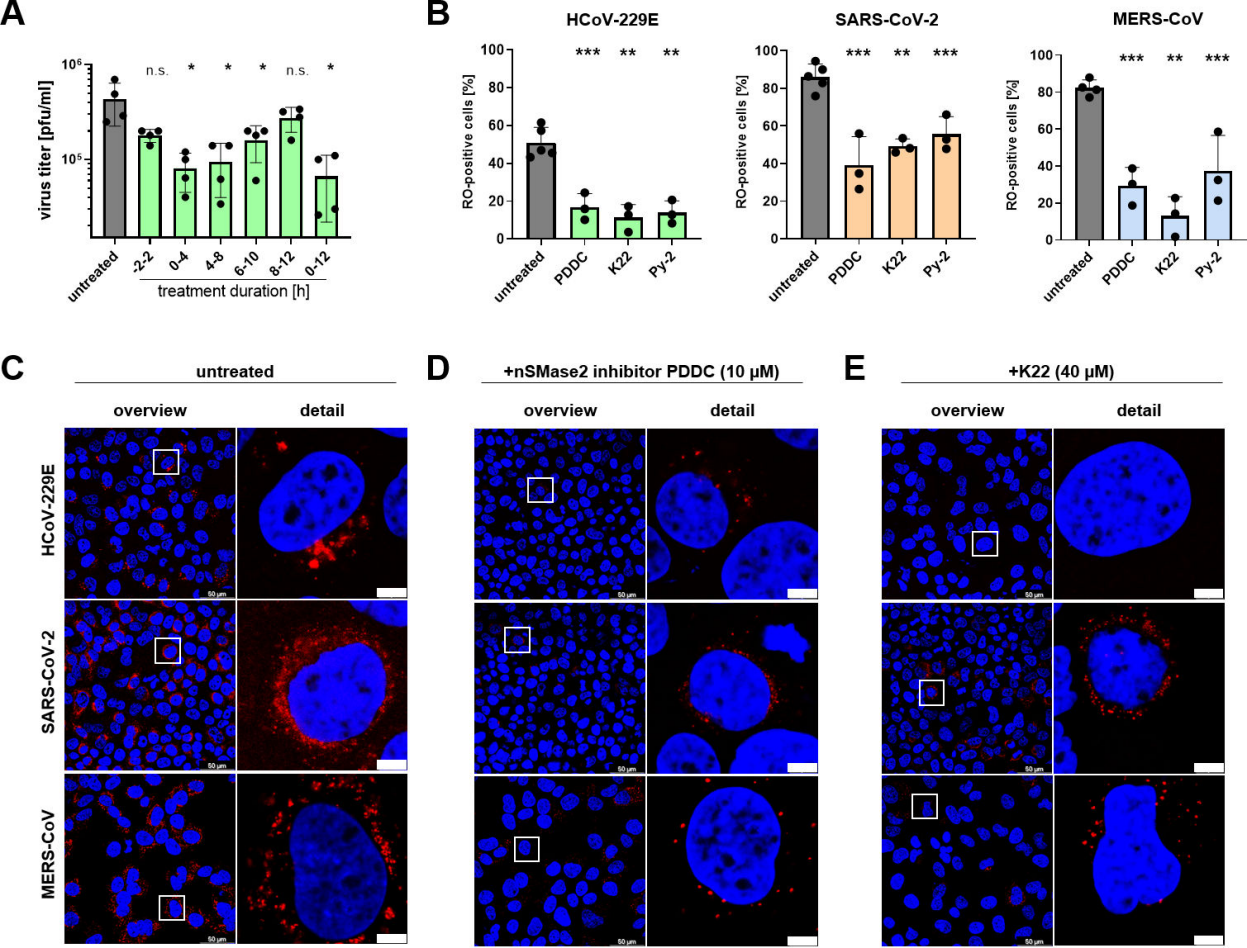
Time-dependent antiviral effects of nSMase2 inhibitor on coronaviral RO formation. (**A**) HCoV-229E-infected Huh-7-ACE2 cells (MOI = 3) were treated with PDDC (10 µM) for different time periods post-infection as indicated below. Production of infectious virus progeny was determined using (pooled) cell culture supernatants collected until 12 hpi. Virus titers were determined and compared to the titer determined for infected but untreated cells. (**B through E**) Huh-7-ACE2 cells were infected with HCoV-229E and then either left untreated (**C**), or treated with PDDC (10 µM, **D**) or K22 (40 µM, **E**) for 8 hpi. Subcellular replication sites were identified by a double-stranded RNA (dsRNA)-specific antibody in the presence or absence of the indicated inhibitor. Nuclei were stained using DAPI. (**B**) Quantification of RO-positive cells by image-based quantification of dsRNA-positive cells in relation to total cell count. All bar graphs show mean ± SD; asterisks indicate *P* values (n.s., not significant; *P < 0.05; **P < 0.005; ***P < 0.0005) obtained by a two-tailed unpaired *t*-test. (**C through E**) Corresponding representative images from one out of three independent experiments. The scale bar in the second row represents 5 µm. All experiments were performed in three independent replicates.

To characterize the presumed role of nSMase2 in more detail, we sought to identify the specific steps in CoV replication that are affected by nSMase2 inhibition. To this end, we performed a time-of-addition experiment (Fig. 3A) in which PDDC (10 µM) was included in the cell culture medium during HCoV-229E adsorption (2 h prior to infection to 2 hpi) or at later time points post-infection (4–8, 6–10, and 8–12 hpi). To determine the total infectious virus progeny produced until 12 hpi (with PDDC added for 4 h at different time points pi), the cell culture supernatants of the respective experiment were collected over time, and the virus titer in the pooled cell culture supernatant was determined by plaque assay. The presence of PDDC in the culture medium between 2 h prior to infection and 2 hpi had no significant effect on the virus titer, indicating that nSMase2 activity has no major role in viral entry. In contrast, the presence of PDDC from 0 to 4, 4 to 8, and 6 to 10 hpi caused a significant reduction of virus titers, while less profound effects were observed if the drug was present in the culture medium later in infection (8–12 hpi; Fig. 3A). The observed time-dependent effects of PDDC on the production of infectious HCoV-229E progeny suggest that nSMase2 activity supports an early step of CoV replication but not the viral entry itself.

Since Cer, the reaction product of nSMase2 activity, is known to redistribute to replication sites of certain +ssRNA viruses (27), which are induced early after viral entry, we next investigated the potential effects of PDDC on coronaviral RO formation in cells infected with HCoV-229E, MERS-CoV, and SARS-CoV-2, respectively (MOI of 3; Fig. 3B through D). For comparison, we also included other known inhibitors of coronaviral RO formation, namely K22 (48, 49) and the cytosolic phospholipase A2a inhibitor Py-2 (50) (Fig. 3B through E; Fig. S2). All three inhibitors diminished the overall number of RO-positive cells compared to the untreated control (Fig. 3B). CoV ROs are known to produce a typical perinuclear staining pattern when analyzed by immunofluorescence microscopy using double-stranded RNA (dsRNA [50]) as a marker for the sites of viral RNA synthesis (Fig. 3C). In the presence of PDDC, the typical punctate perinuclear staining pattern was significantly diminished (Fig. 3D). The reduction was comparable to the effect of the known RO formation inhibitors K22 (Fig. 3E) and Py-2 (Fig. S2).

Taken together, the data lead us to suggest that nSMase2 activity might play a role in the process of RO formation occurring in the first few hours post-infection but has a less critical role at later stages of the viral replication cycle in Huh-7-ACE2 cells.

### nSMase2 and its product ceramide, but not sphingomyelin, colocalize with CoV-induced replication organelles

**Fig 4:**
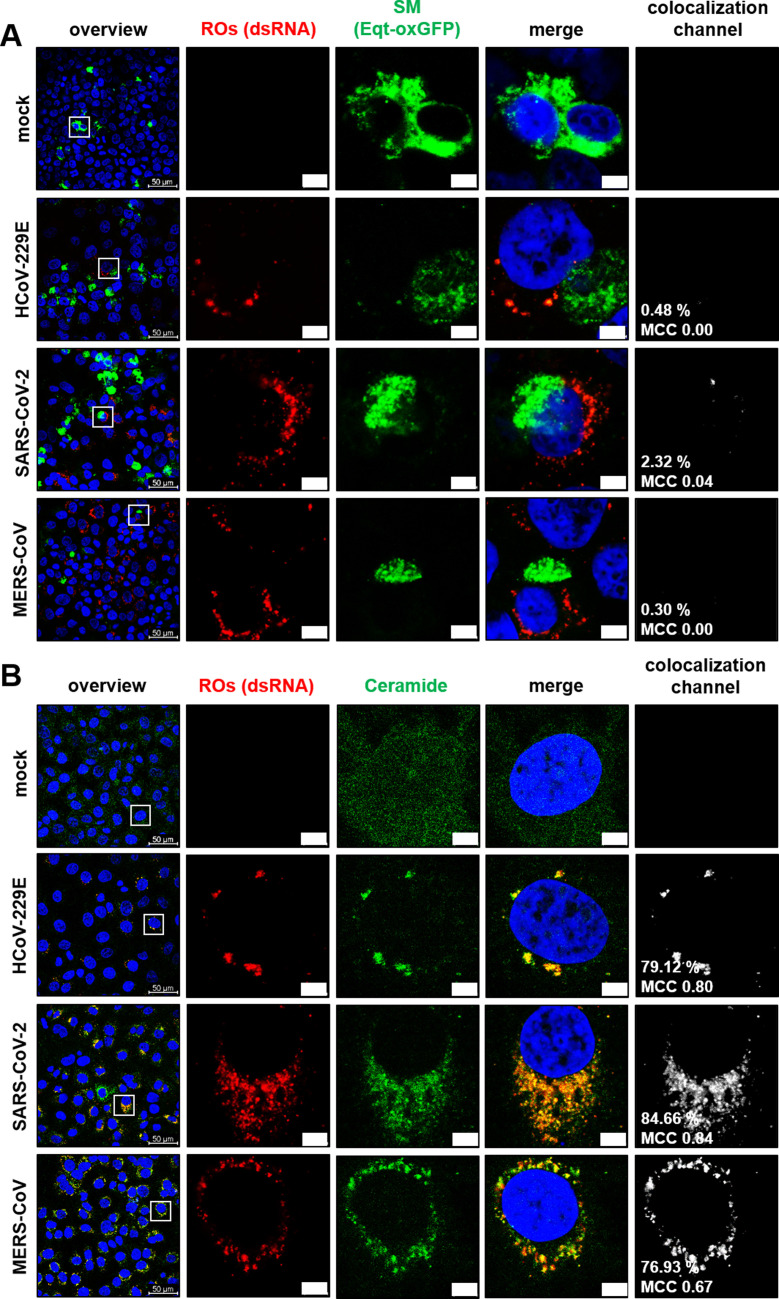
Colocalization of ROs and sphingolipids in CoV-infected cells. (**A**) Huh-7-ACE2 cells were transfected with an Eqt-SM-oxGFP expression construct (to visualize SM, green) and after 48 h infected with the indicated CoV (MOI = 3). Eight hours post-infection, cells were fixed and stained for viral ROs (red) using an antibody against dsRNA, a specific marker for viral replication intermediates. (**B**) Huh-7-ACE2 cells were infected with the indicated CoV (MOI = 3) for 8 hpi, fixed, and permeabilized using 0.5% saponin. Cells were stained for viral ROs (dsRNA, red) using an antibody against dsRNA and an antibody against Cer (green). DAPI was used for staining of nuclei. Insets indicate regions of interest displayed at higher magnification in the next row. Colocalization signals, rates, and Manders correlation coefficients (MCCs) were calculated for the total images. Scale bars = 5 µm. Representative images from one out of three biologically independent experiments were shown.

To further corroborate the potential role of nSMase2 and its product Cer in RO formation, we investigated a possible colocalization of sphingolipids and viral ROs. To visualize the intracellular distribution of SM in CoV-infected cells, we utilized Eqt-SM-oxGFP, a plasmid DNA-encoded fusion protein construct that is capable of binding diverse pools of SMs (51, 52). We did not observe any colocalization of Eqt-SM-oxGFP and viral ROs, indicating that SMs do not accumulate at the site of viral RNA synthesis at 8 hpi (Fig. 4A). In contrast, endogenous Cer (as visualized using a commercially available antibody) was confirmed to colocalize with ROs of all three CoVs, with the typical clustering in the perinuclear region of infected cells (Fig. 4B).

Having confirmed a significant overlap of the Cer signal with sites of viral ROs, we addressed the question of whether nSMase2, which hydrolyzes SM to Cer, can be detected in close proximity to viral ROs. Because commercially available nSMase antibodies are known to broadly cross-react in Western blot analyses, making them largely unsuitable for specific detection (53), we resorted to analyzing the nSMase2 distribution by transfection of an expression construct encoding the human nSMase2 fused to eGFP (54).

**Fig 5:**
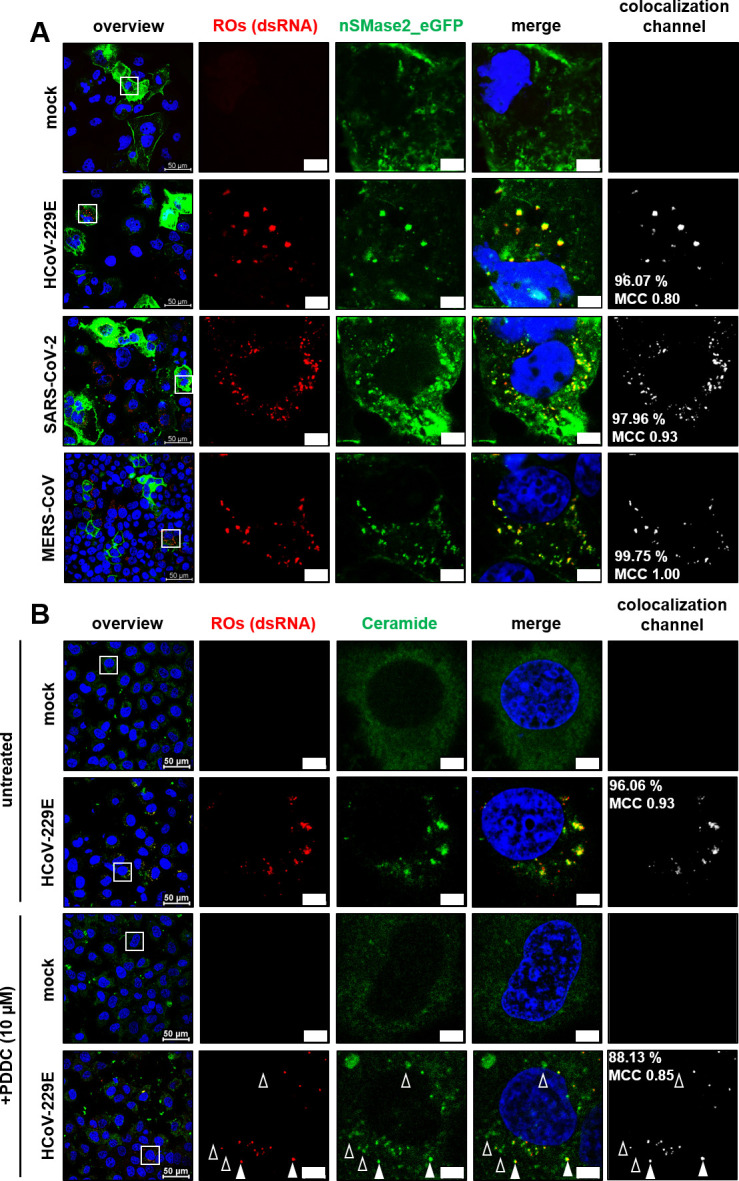
Colocalization of CoV-induced ROs with nSMase2. (**A**) Huh-7-ACE2 cells were transfected with an nSMase2-eGFP-expressing construct (to visualize sphingomyelinase, green) and infected with HCoV-229E, SARS-CoV-2, or MERS-CoV (MOI = 3) and fixed 8 hpi with 3.7% paraformaldehyde (PFA). Viral ROs (red) were stained using an antibody against dsRNA. (**B**) Huh-7-ACE2 cells were infected with HCoV-229E (MOI = 3) and treated as indicated with the nSMase2 inhibitor PDDC. Viral ROs (red) and ceramide (green) were stained using respective antibodies. Filled arrows indicate colocalization. Outline arrows indicate ceramide spots without a dsRNA signal. DAPI was used for staining of nuclei. Insets indicate regions of interest displayed at higher magnification in the next row. Colocalization signals, rates, and Manders correlation coefficients (MCCs) were calculated for the total images. Scale bars = 5 µm. Representative images from one out of three biologically independent experiments were shown.

As shown before for Cer, nSMase2-eGFP was found to colocalize with ROs induced in cells infected with HCoV-229E, MERS-CoV, and SARS-CoV-2, respectively, at 8 hpi (Fig. 5A). In contrast, overexpression of eGFP using the pcDNA3.1-eGFP construct did not lead to prominent colocalization (Fig. S3). Similarly, a catalytically inactive form of nSMase2-eGFP mutant harboring an active-site substitution (H639A [55]) colocalized with viral ROs, suggesting that the potential recruitment of nSMase2 to sites of viral RO accumulation is independent of enzymatic activity (Fig. S4).

Finally, we examined the recruitment of Cer species following inhibition of nSMase2 activity by PDDC in HCoV-229E-infected cells (Fig. 5B). Compared to untreated cells, the number of dsRNA-positive cells was drastically diminished, whereas the intensity of the Cer signal seemed to be largely unaffected. All dsRNA-positive sites were also positive for Cer (Fig. 5B, filled arrows), suggesting that Cer was produced at (and/or partially redistributed to) the few remaining ROs being formed, possibly by using alternative Cer biosynthesis pathways.

Based on these data, it seems reasonable to suggest that Cer species represent major lipid constituents of ROs produced in CoV-infected Huh-7-ACE2 cells, and their synthesis involves nSMase2 activities concentrated at these sites, although additional Cer synthesis pathways fueling potential RO formation cannot be excluded at this stage.

### Artificially induced replication organelles deregulate sphingolipid homeostasis

To provide additional support for the proposed critical role of Cer and nSMase2 in RO formation, we employed a surrogate system to produce RO-like structures independent of viral infection. As previously shown for several betacoronaviruses, the production of nsp3 and nsp4 suffices to induce the formation of RO-like structures in transfected cells (22, 23, 25, 56). A plasmid encoding the nsp3-4 construct that undergoes autoproteolytic cleavage by the papain-like protease (PL^pro^) residing in nsp3 can be used to characterize these artificially induced membrane structures (23, 26).

**Fig 6:**
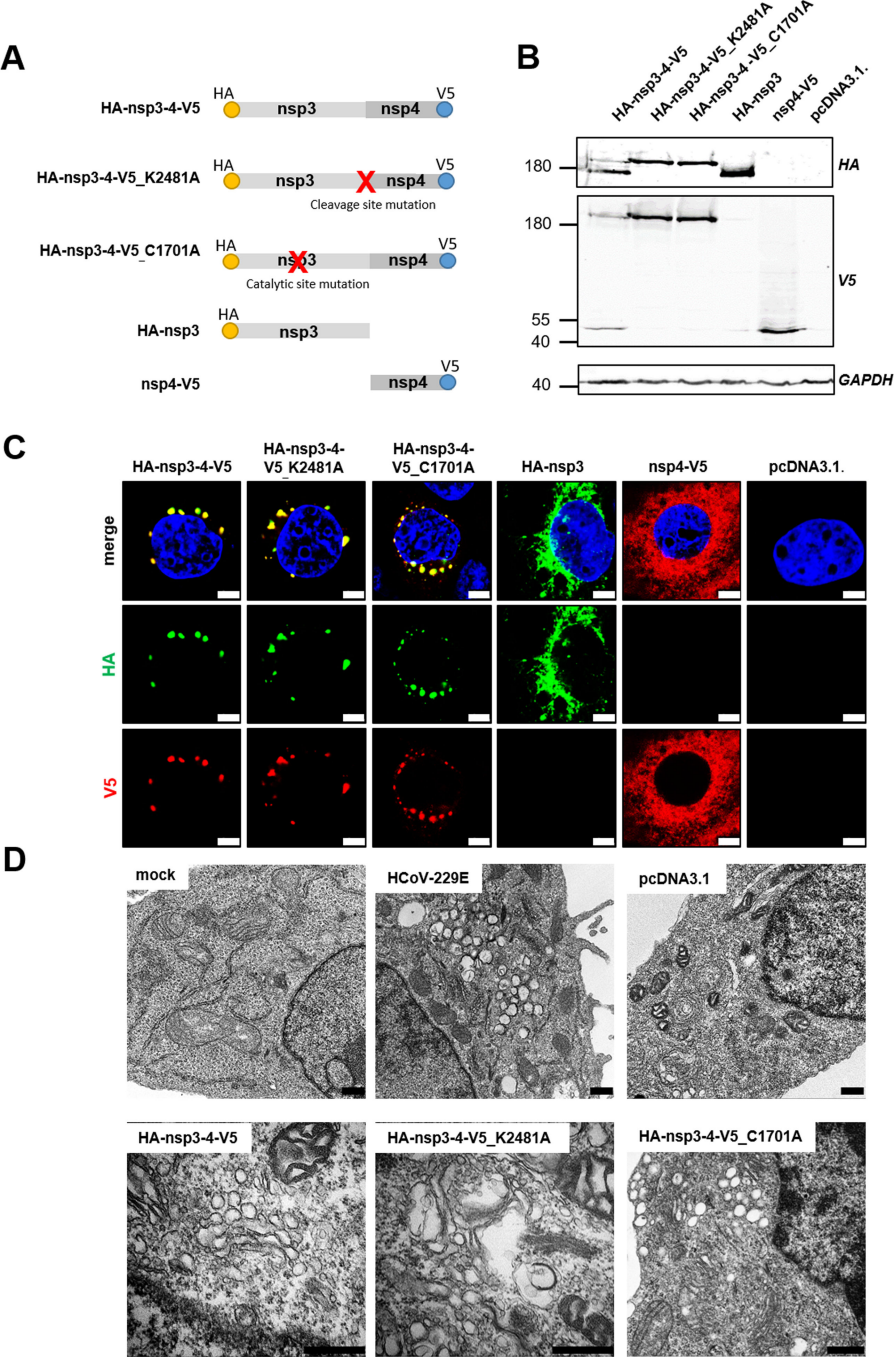
Artificially induced ROs by overexpressing a self-cleaving HCoV-229E nsp3-4 construct. (**A**) Schematic illustration of constructs generated to induce artificial HCoV-229E ROs upon transfection. The epitope tags used at the termini of the constructs are indicated as dots. The HA-nsp3-4-V5_K2481A construct contains an alanine substitution in the cleavage site of nsp3-4, therefore avoiding nsp3-mediated polyprotein cleavage. The HA-nsp3-4-V5_C1701A construct contains an alanine substitution that abrogates PL^pro^ activity. (**B**) HEK-293T-ACE2 cells were transfected for 24 h with the indicated expression constructs, lysed, and HA-nsp3 and nsp4-V5-tagged proteins were detected using Western blot analysis. GAPDH served as a loading control. (**C**) Huh-7-ACE2 cells were transfected for 24 h with the indicated expression constructs, fixed, and stained using HA-specific (green) or V5-specific (red) antibodies. Subcellular localization was visualized by confocal microscopy using a Leica SP05. DAPI was used for staining of nuclei. Scale bars = 5 µm. (**D**) Huh-7-ACE2 cells were transfected with the indicated constructs, fixed 24 hours post-transfection, and analyzed via transmission electron microscopy analysis using a Zeiss LEO electron microscope. Scale bars = 500 nm.

Here, we sought to adopt this system to artificially induce alphacoronavirus RO-like structures using HCoV-229E as a representative of this coronavirus genus (Fig. 6). To this end, we used different plasmid DNA constructs that were codon optimized for expression in human cells. The proteins encoded by these constructs carried hemagglutinin (HA) or V5 epitopes at their termini to allow their specific detection by appropriate monoclonal antibodies (Fig. 6A).

Following transfection of HEK-293T-ACE2 cells with these expression constructs, viral protein expression and proteolytic cleavage at the nsp3|nsp4 site were confirmed by Western blot analysis (Fig. 6B). The HA-nsp3-4-V5 fusion protein construct was nearly completely cleaved into mature nsp3 (~170 kDa) and nsp4 (~50 kDa). We also generated mutant forms of HA-nsp3-4-V5 in which either the conserved Lys residue at the P4 position of the nsp3|nsp4 cleavage site was replaced with Ala (HA-nsp3-4-V5_K2481A) or the active-site Cys residue of the PL^pro^ domain in nsp3 was replaced with Ala (HA-nsp3-4-V5_C1701A). As expected, both mutant constructs resulted in the production of a single (uncleaved) protein that co-migrated with the remaining full-length protein HA-nsp3-4-V5 (Fig. 6B, compare lane 1 with lanes 2 and 3). In addition, we were able to confirm the identities of the respective N- and C-terminal cleavage products, HA-nsp3 (~170 kDa) and nsp4-V5 (~50 kDa). The identities of the proteins released by proteolytic cleavage from HA-nsp3-4-V5 were also confirmed by their comigration in SDS-polyacrylamice gels with HA-nsp3 and nsp4-V5 expressed from other plasmid constructs (Fig. 6B, lane 4 and 5).

Next, we evaluated the subcellular localization of nsp3 and nsp4 in Huh-7-ACE2 cells by immunofluorescence using HA- and V5-tag-specific antibodies (Fig. 6C). In cells expressing HA-nsp3-4-V5 and the two mutant derivatives of HA-nsp3-4-V5 (HA-nsp3-4-V5_K2481A and HA-nsp3-4-V5_C1701A), we consistently observed the typical perinuclear staining pattern (as shown before for viral ROs; Fig. 3) with colocalization of HA-nsp3 and nsp4-V5, suggesting that abolished proteolytic cleavage at the nsp3|nsp4 site does not impair proper subcellular localization of the uncleaved protein. In striking contrast, when expressed individually, nsp3 and nsp4 displayed a cytoplasmic, partially reticular staining pattern (Fig. 6C), suggesting that both proteins are required to induce the formation of RO(-like) structures resembling those detectable in virus-infected cells.

To further investigate the membrane remodeling induced by nsp3 and nsp4 via membrane zippering (56, 57), we performed electron microscopy to resolve membrane structures in cells expressing HA-nsp3-4-V5 or one of the mutant constructs HA-nsp3-4-V5_K2481A and HA-nsp3-4-V5_C1701A (Fig. 6D). Similar to virus-induced ROs, the expression and proteolytic processing of HA-nsp3-4-V5 led to extensive remodeling of cellular membranes with the formation of typical double membrane structures in the perinuclear region (Fig. 6D), as demonstrated previously for betacoronaviruses (23, 25, 26). Interestingly, the expression of proteolytically inactive forms of HA-nsp3-4-V5 (HA-nsp3-4-V5_K2481A and HA-nsp3-4-V5_C1701A) also led to membrane zippering and the occurrence of vesicular structures similar to the structures observed in HA-nsp3-4-V5 expressing cells.

### Artificially induced replication organelles colocalize with cellular ceramides

**Fig 7:**
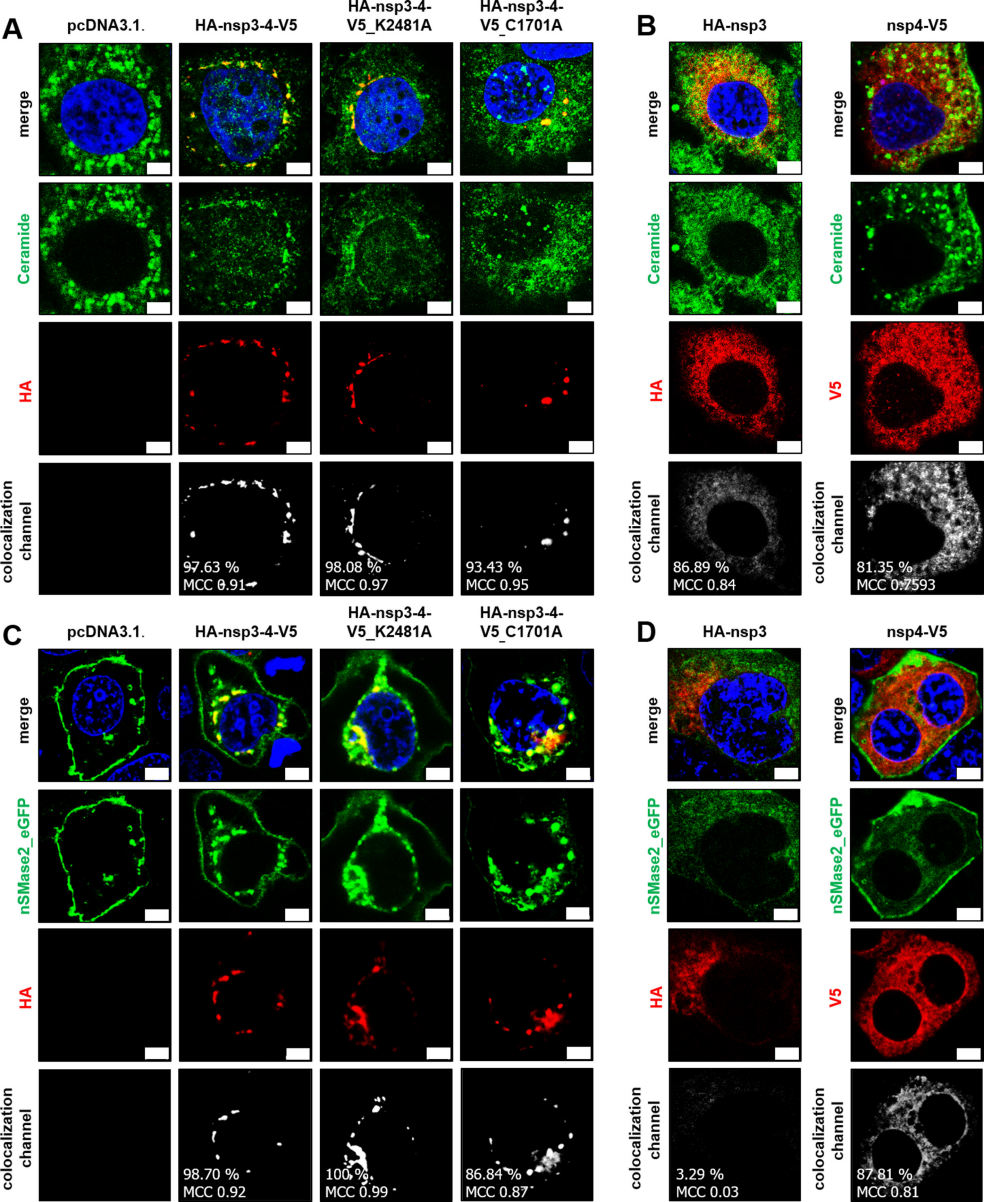
Colocalization of artificially induced ROs and Cer. (**A and B**) Huh-7-ACE2 cells were transfected with the indicated plasmids (0.75 µg DNA) expressing either HA-nsp3-4-V5 or mutants (HA-nsp3-4-V5_K2481A and HA-nsp3-4-V5_C1701A) (**A**) or the single constructs (**B**). After 24 h, the cells were fixed with 3.7% paraformaldehyde (PFA). The cells were then permeabilized with 0.5% saponin. Nsp3 or 4 (red) and Cer (green) were visualized using HA- (nsp3), V5- (nsp4), and Cer- specific antibodies. (**C and D**) Huh-7-ACE2 cells were transfected with the indicated plasmids (0.75 µg DNA) expressing nSMase_eGFP (green) and either HA-nsp3-4-V5 or mutants (HA-nsp3-4-V5_K2481A and HA-nsp3-4-V5_C1701A; **C**) or the single constructs (**D**). After 24 h, the cells were fixed with 3.7% PFA. The cells were then permeabilized with 0.5% saponin. Nsp3 or 4 (red) visualized using HA- (nsp3) or V5- (nsp4) specific antibodies. DAPI was used for staining of nuclei. Colocalization signals, rates, and Manders correlation coefficients (MCCs) were calculated for the total images. Representative images from one out of three biologically independent experiments were shown. Scale bars = 5 µm.

Using the nsp3-4 expression system, we performed a colocalization study of Cer and nSMase2-eGFP in cells transfected with either the nsp3-4-encoding constructs or a construct expressing only one of these proteins, nsp3 or nsp4 (Fig. 7). Strikingly, Cer (Fig. 7A) and nSMase2-eGFP (Fig. 7C) colocalized with HA-nsp3-4-V5 as well as the two HA-nsp3-4-V5 mutants. Again, nSMase2-eGFP and its product Cer were found to be enriched in areas showing nsp3-4-induced membrane rearrangements. In contrast, there was much less local enrichment and colocalization of Cer with nsp3 or nsp4, if only one of the two proteins was produced in these cells (Fig. 7B and D). Therefore, we assume Cer and nSMase2 involvement in the formation of artificially induced RO-like structures upon transfection with HA-nsp3-4-V5, irrespective of polyprotein cleavage.

**Fig 8:**
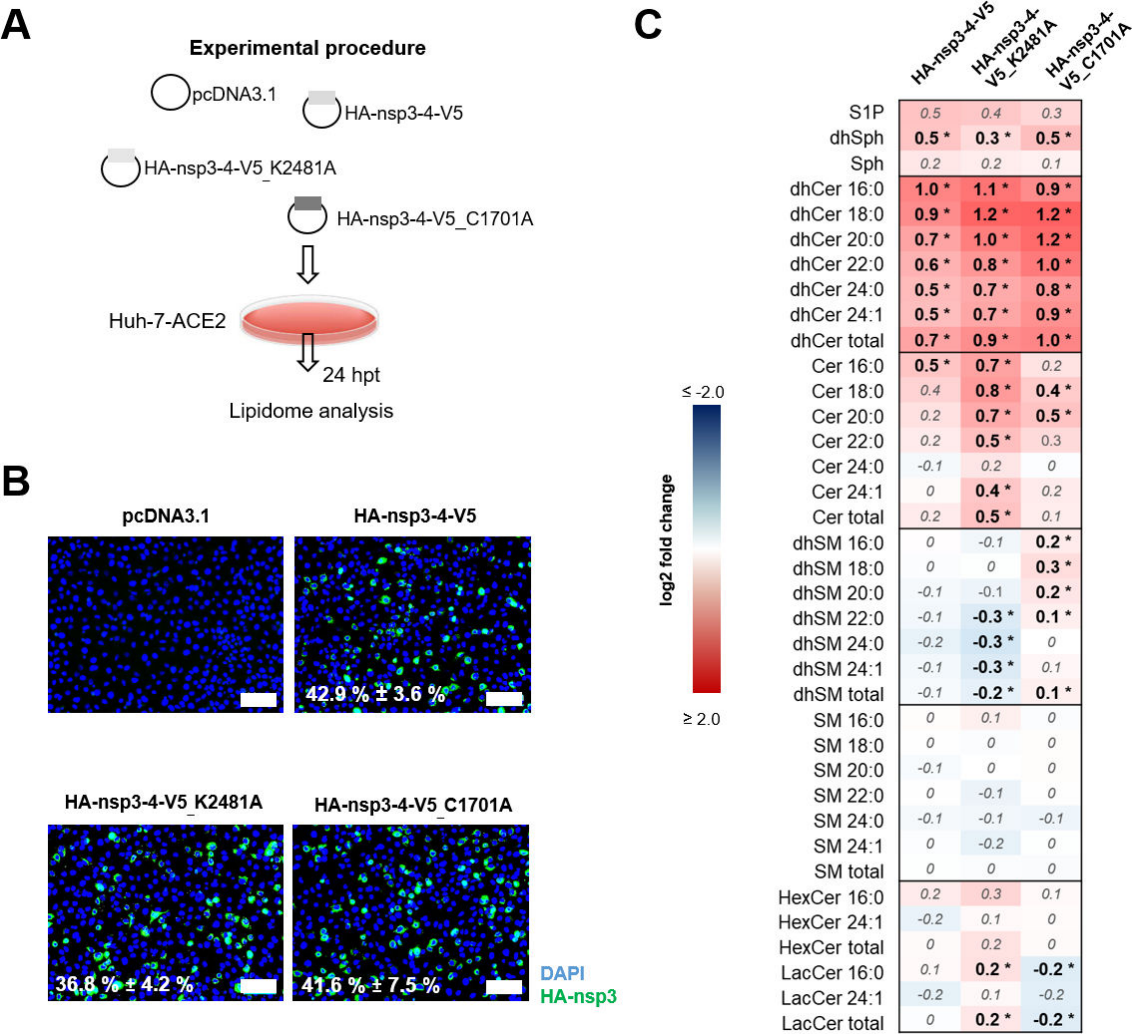
Overview of sphingolipid (SL) changes upon artificial RO formation upon transfection with constructs expressing nsp3 and nsp4 in Huh-7-ACE2 cells. (A) Experimental design of the sphingolipidome analysis. Huh-7-ACE2 cells were mock transfected with empty vector control (pcDNA3.1) or transfected with either HA-nsp3-4-V5, HA-nsp3-4-V5_K2481A or HA-nsp3-4-V5_C1701A for 24 h. (B) Corresponding immunofluorescence images of transfected cells. The value indicates transfection efficacy of HA-nsp3-positive cells (green) in relation to total cell count. Scale bars = 100 µm. (C) Heatmap showing fold changes of deregulated SL species in relation to mock-transfected control (significant differences in bold and marked with asterisks, *P* ≤ 0.05). Cer, ceramide; dhCer, dihydroceramide; dhSM, dihydrosphingomyelin; dhSph, dihydrosphingosine; HexCer, hexosylceramide; LacCer, lactosylceramide; S1P, sphingosine-1-phosphate; SL, sphingolipid; SM, sphingomyelin; Sph, sphingosine

To verify a potential involvement of Cer also in ROs induced by the nsp3-4 expression system, we examined whether the expression and cleavage of nsp3-4, along with the resulting membrane alterations resembling RO-like structures, affect global sphingolipid metabolism similarly to that observed during infection. For this, we performed a second lipidome analysis of Huh-7-ACE2 cells transfected with an empty vector DNA (pcDNA3.1), HA-nsp3-4-V5, or one of the two HA-nsp3-4-V5 mutant constructs (Fig. 8A). To ensure comparable transfection efficiency for lipid analysis, we conducted immunofluorescence and confirmed equal transfection rates of approximately 40% (Fig. 8B). The values obtained for individual sphingolipid species were then compared to those obtained for cells transfected with the empty vector DNA (pcDNA3.1) to rule out possible effects of the transfection procedure on the cellular lipid content and composition. Consistent with the data shown in Fig. 1 for CoV-infected cells, the transfection of HA-nsp3-4-V5 (inducing artificial ROs) led to a slight but significant upregulation of dhCer and the most abundant Cer species (Cer16:0; Fig. 8C). Interestingly, the two HA-nsp3-4-V5 mutant constructs, which were shown to induce similarly profound membrane rearrangements in transfected cells (Fig. 6), induced similar sphingolipid deregulations, further supporting the idea that co-expression of nsp3 and nsp4 (but not their proteolytic release from precursor proteins) is required to cause the observed membrane rearrangements and the associated changes in sphingolipid metabolism.

Overall, the observed deregulation of sphingolipids was not as prominent as shown before for CoV-infected cells, which may be due to (i) lower transfection rates compared to the high-multiplicity infection rates used in earlier experiments, (ii) time dependencies in RO formation upon infection or transfection, or (iii) the absence of nsp6, which was suggested to play a supportive role in RO formation (58).

### Ceramide colocalizes with coronaviral replication organelles in lung-derived cell systems

**Fig 9:**
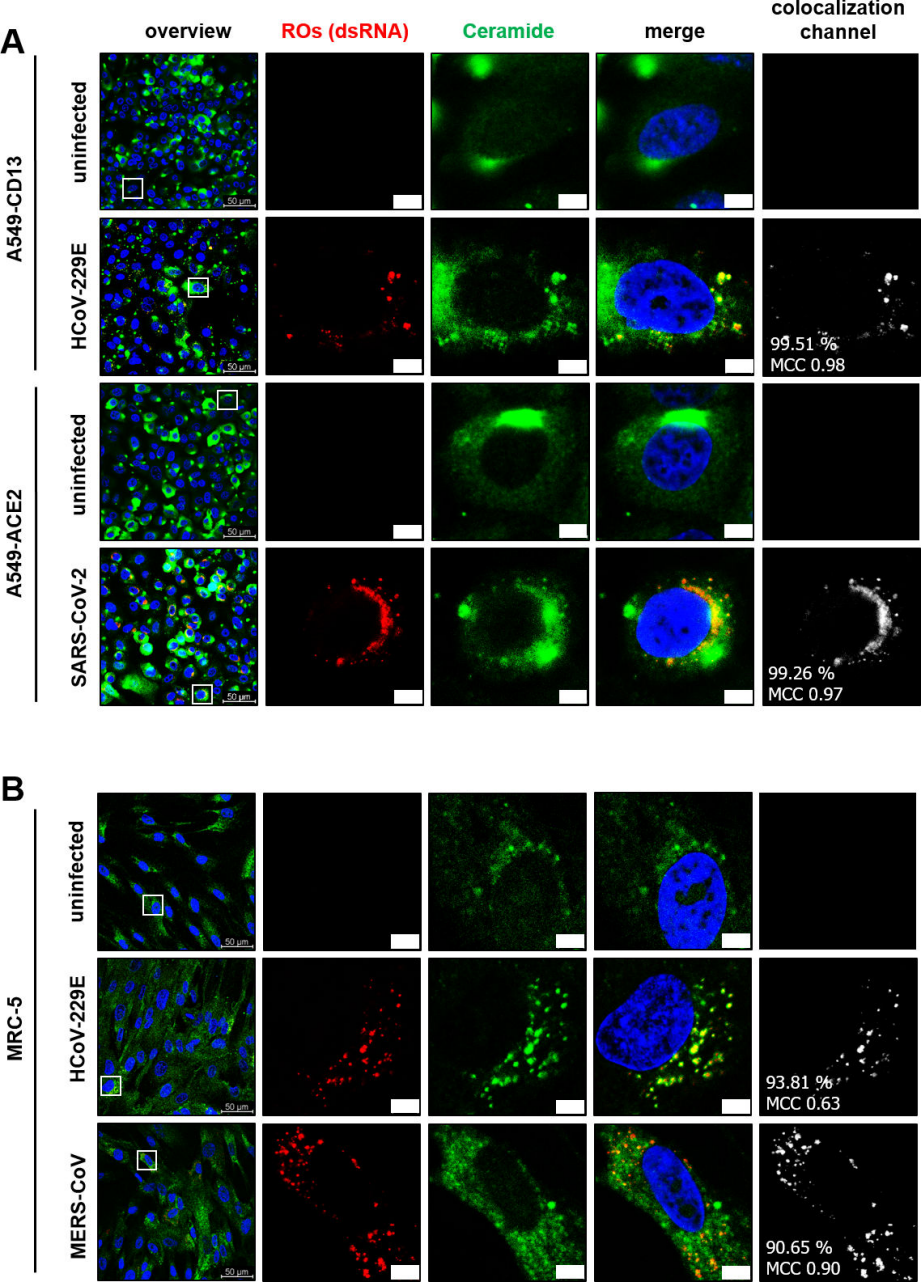
Colocalization of CoV-induced ROs and Cer in lung-derived cells. (A) Adenocarcinoma cell line A549-ACE2 (for SARS-CoV-2) or A549-CD13 (for HCoV-229E) or (B) primary lung fibroblasts MRC-5 cells (for HCoV-229E and MERS-CoV) were infected with an MOI of 3 for 8 hpi. The fixed cells were then permeabilized with 0.5% saponin and stained against dsRNA (red) and Cer (green). DAPI was used for staining of nuclei. Colocalization signals, rates and MCCs were calculated for the total images. Scale bars = 5 µm. Representative images from one out of three biologically independent experiments were shown.

The data presented above provided strong evidence for the involvement of Cer and nSMase2 in the formation of coronaviral ROs in Huh-7-ACE2 cells. We next addressed the question of whether this CoV-induced sphingolipid deregulation also applies to other cell types, focusing on more relevant, lung-derived cells susceptible to CoVs (Fig. 9). For SARS-CoV-2, we used A549 cells overexpressing ACE2 (A549-ACE2), a lung-derived adenocarcinoma cell line (Fig. 9A). For HCoV-229E, we used A549 cells overexpressing CD13 and TMPRSS2 (A549-CD13) (59). In addition, we used embryonal lung fibroblasts (MRC-5 cells) for infections with HCoV-229E and MERS-CoV, respectively. In line with previous results for Huh-7-ACE2 cells, the data shown in Fig. 9A and B indicate colocalization of viral ROs with endogenous Cer in CoV-infected lung-derived cell culture systems, albeit with a less profound redistribution of Cer compared to Huh-7-ACE2 cells.

### Comparable cell-type sphingolipid deregulation upon CoV infection in lung-derived cell systems

**Fig 10:**
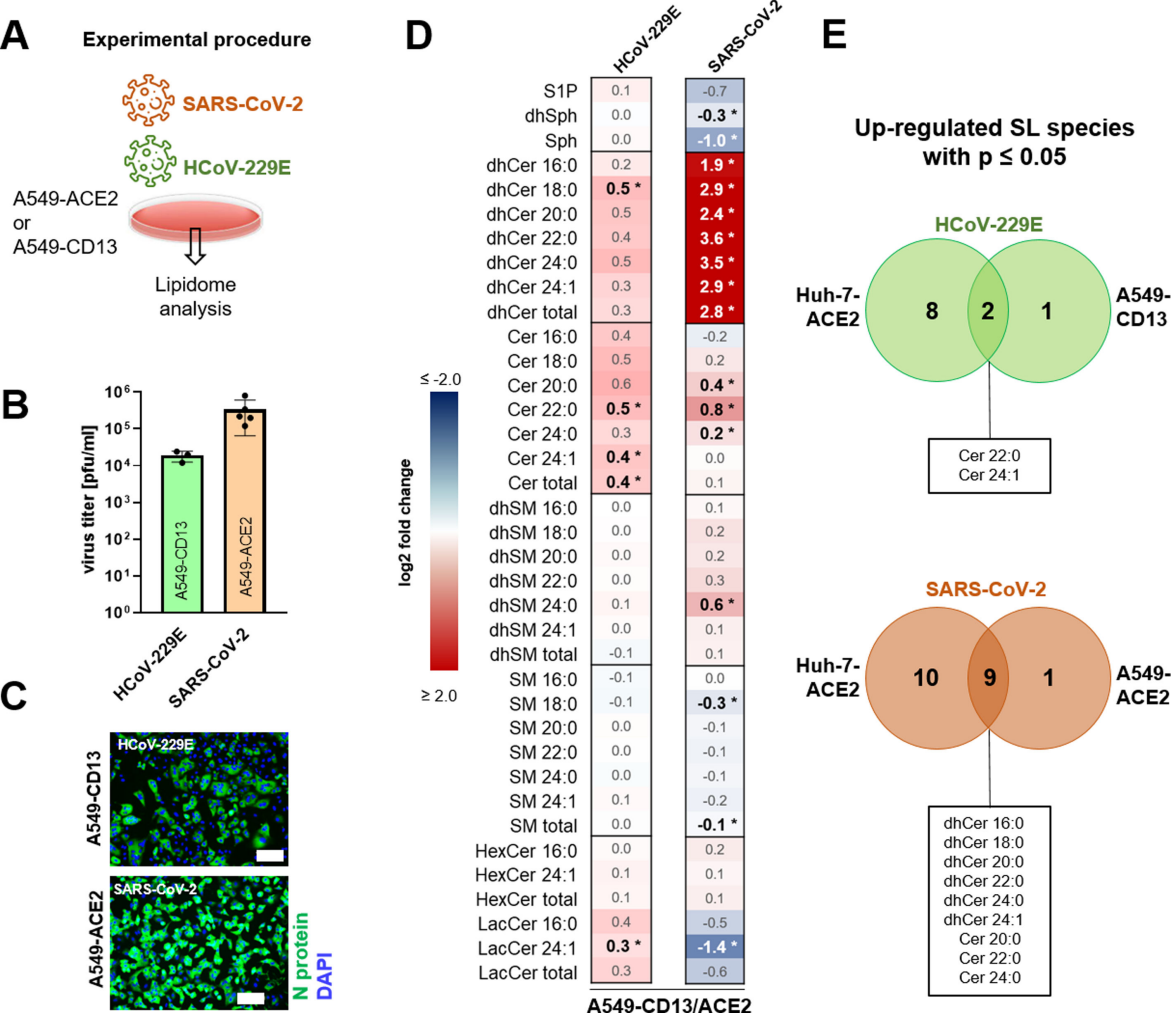
Overview of sphingolipid changes upon infection with HCoV-229E and SARS-CoV-2 in lung-derived cells. (A) Experimental design of the sphingolipidome analysis. A549-ACE2 or A549-CD13 cells were mock-infected or infected with HCoV-229E (A549-CD13) or SARS-CoV-2 (A549-ACE2) with an MOI of 3 for 12 hpi. (B and C) Corresponding viral titers and immunofluorescence images of A549-ACE2 (for SARS-CoV-2) and A549-CD13 (for HCoV-229E) cells (MOI = 3) 12 hpi. Scale bars = 100 µm. (D) Heatmap showing fold changes of deregulated sphingolipid species at the indicated time points in relation to uninfected control based on significant differences (significant differences in bold and marked with asterisks, *P* ≤ 0.05) calculated from the replicates by *t*-test (SARS-CoV-2) or one-way ANOVA with Dunnett´s test for multiple comparisons (HCoV-229E). (E) Corresponding Venn diagrams. Experiments were done in biological independent replicates (*n *= 5). Cer, ceramide; dhCer, dihydroceramide; dhSM, dihydrosphingomyelin; dhSph, dihydrosphingosine; HexCer, hexosylceramide; LacCer, lactosylceramide; S1P, sphingosine-1-phosphate; SL, sphingolipid; SM, sphingomyelin; Sph, sphingosine.

In a final series of experiments, we expanded our lipidome analysis to the lung-derived cell culture systems described above (Fig. 10A). To ensure comparable viral replication characteristics in the different cell systems used, viral titers and infection rates were determined at 12 hpi (Fig. 10B and C). HCoV-229E replicated to slightly lower titers (~10^4^ pfu/mL) in A549-CD13, while SARS-CoV-2 replicated to titers of ~10^5^ pfu/mL in A549-ACE2 cells at this time point pi (Fig. 10B). Most likely, the lower titers observed for HCoV-229E were due to a lower infection rate of HCoV-229E in these cells (Fig. 10C). The data obtained in the subsequent sphingolipidome analysis are summarized in Fig. 10D.

For HCoV-229E-infected A549-CD13 cells and SARS-CoV-2-infected A549-ACE2, we found a deregulation of sphingolipids that were similar to that observed for CoV-infected Huh-7-ACE2 cells, albeit with less profound changes in most cases. For HCoV-229E, the Cer22:0, Cer24:1, and total Cer levels were significantly increased in Huh-7-ACE2- and A549-CD13-infected cells (Fig. 10E). For SARS-CoV-2, all dhCers, Cer20:0, Cer22:0, and Cer24:0 levels were significantly increased (Fig. 10E), whereas SM18:0 was downregulated in Huh-7-ACE2 and A549-ACE2 cells. As indicated above, the sphingolipid deregulations in CoV-infected A549-derived cells were less profound than those observed in Huh-7-ACE2 cells. It remains to be studied if this is due to the somewhat lower infection rate and/or replication efficiency (for HCoV-229E) or is linked to cell type-specific differences.

## DISCUSSION

Following the SARS-CoV-2 pandemic, systems biology approaches have significantly enhanced our understanding of the interactions between the virus and the host (60). While these studies are mainly limited to SARS-CoV-2, other CoVs have been studied less extensively, and information on host factors that are essential for the replication of genetically diverged CoVs from different genera remains limited, complicating the identification of CoV-wide conserved factors and mechanisms that may be employed as targets in the development of broadly acting antiviral drugs.

In an effort to fill these knowledge gaps, we performed a comparative lipidome analysis using three different human pathogenic CoVs. We found that all three CoVs cause major changes in the cellular sphingolipid metabolism of infected Huh-7-ACE2 cells, including a strong increase of Cer species and a decrease of SMs. Similar changes in the Cer and SM content were observed in SARS-CoV-infected A549-ACE2 and HCoV-229E-infected A549-CD13 cells.

The data are largely consistent with previous studies. Thus, increased Cer levels have also been reported for other +ssRNA viruses known to trigger intracellular membrane rearrangements, such as tick-borne encephalitis virus (61), Sindbis virus (62), and Dengue virus (63). For CoVs, elevated Cer levels were previously reported for HCoV-229E-infected Huh-7 cells (50), MERS-CoV-infected Calu-3 cells (64), and SARS-CoV-2-infected HEK-293T-ACE2, A549-ACE2 (60), and VeroE6 cells (65, 66), suggesting a conserved and cell type-independent change of cellular sphingolipids in CoV-infected cells.

There is now a large body of evidence to suggest the biological significance of the observed upregulation of Cer and downregulation of SM in infected cells. More specifically, the available data support the conclusion that Cer species and SMases, the key enzymes catalyzing the conversion of SM to Cer, have important functions in viral replication in the vast majority of cell types studied to date.

Interestingly, our study revealed that aSMase supports the replication of SARS-CoV-2 but has no major role in MERS-CoV or HCoV-229E replication. Earlier studies using vesicular stomatitis virus (VSV) pseudoviral particles containing the SARS-CoV-2 spike protein showed a translocation of aSMase to the plasma membrane, leading to SM hydrolysis and thereby facilitating vesiculation and endocytotic uptake (67). Moreover, pharmacological and genetic aSMase inhibition was shown to reduce VSV-SARS-CoV-2 spike entry in permanent and primary cells (36). It remains to be investigated whether aSMase activity is specifically required for SARS-CoV-2 or also for other CoVs, such as SARS-CoV and HCoV-NL63, that both employ ACE2 as a major entry receptor (68).

In contrast to aSMase, nSMases (especially nSMase2) appear to be critically involved in the replication of all three CoVs in Huh-7-ACE2 cells. Inhibition of nSMase2 activity or reduced expression levels of this enzyme caused a reduction of viral titers and affected the formation of viral ROs, whereas viral entry remained unaffected. Strikingly, nSMase2 and its product Cer, but not SM, colocalized with infection-induced or artificially induced ROs in Huh-7-ACE2 cells. Cer also colocalized with coronaviral ROs in other lung-derived cell systems, including embryonic lung fibroblasts (MRC-5 cells).

Taken together, the data lead us to suggest that Cer species, irrespective of whether they are recruited from existing cellular deposits or newly generated nSMase2-dependent or other metabolic pathways, are integral components of coronaviral ROs. This conclusion is consistent with earlier studies reporting Cer accumulation at the replication sites or specialized replication organelles of other +ssRNA viruses. For example, Cer was shown to redistribute to the Zika virus (ZIKV) and West Nile virus (WNV) replication sites (27, 28), and nSMase2 inhibition was revealed to reduce ZIKV and WNV replication (69, 70). The present study extends these observations to genetically diverse CoVs representing different genera, and it provides strong support for a major role of Cer accumulation that likely contributes to efficient formation of ROs in infected cells, thereby providing a structural platform at which viral RNA synthesis is orchestrated by the viral replication-transcription complex.

In general, changes in the lipid composition of membranes are associated with membrane curvature and vesiculation (71). According to the bilayer couple hypothesis (72), the two leaflets of a lipid bilayer are tightly coupled, with asymmetric changes in one leaflet having the potential to induce significant structural changes, such as membrane bending, fission, and fusion (73–75). Thus, for example, asymmetric cleavage of sphingolipids in a lipid bilayer by SMases (the latter converting cylindrical SMs into cone-shaped Cer) can be expected to induce spontaneous negative membrane curvature and vesiculation potentially involved in RO formation. Moreover, Cer-enriched microdomains might promote domain-induced budding (76, 77) or the recruitment of membrane-bending proteins (78).

An example of how Cer and nSMase2 influence membrane curvature and vesiculation is the process of exosome formation (79–82). Cer species are highly enriched in purified exosomes, and inhibition of nSMase2 activity was shown to impair the release of exosomes. Also, artificial vesicle budding can be induced by exogenous nSMase2 added to giant unilamellar vesicles consisting of dioleoylphosphatidylcholine, SM, and cholesterol (82). Moreover, Cer and nSMase2 are thought to regulate other vesiculation-dependent processes like endocytosis (83, 84)

In Huh-7-ACE2 cells, the generation of Cer upon infection seems to be nSMase2 dependent. A possible mechanism might be the recruitment of nSMase2 by neutral sphingomyelinase activation associated factor (NSMAF, also called FAN) early after infection, enhancing Cer production by activating nSMase2 (85) at LC3-positive EDEMosomes (85–88). LC3-positive EDEMosomes are believed to be linked to viral RO formation since SARS-CoV- and murine coronavirus (MHV)-induced ROs colocalize with LC3 (89, 90).

It is tempting to speculate that NSMAF and/or nSMase2 are recruited to membrane rearrangement sites by specific interactions with viral nsp3 or nsp4 or other currently unknown interaction partners. To our knowledge, only nSMase3 has been identified as a direct interaction partner for SARS-CoV-2 nsp3 (91) and has also been found in close proximity to SARS-CoV-2 nsp4 (92). Since antibodies that are suitable to specifically detect nSMases and NSMAF are currently not available, proximity labeling studies, as done before for MHV (93), may provide an appropriate approach to validate a potential nSMase2 (or NSMAF) recruitment and activation mechanism at the site of viral RO formation in different cell types.

To further validate the proposed role of Cer in RO formation, it would be desirable to perform lipidomic analysis using purified ROs isolated from cells, as previously done for hepatitis C virus (HCV) (94). Ideally, these studies should also include more complex cell systems with different cell types to get more insight into cell type-specific requirements for sphingolipids and the cellular enzymes involved in their production to generate the ROs required for efficient CoV replication.

Potentially, these studies may also provide new avenues for therapeutic intervention. A range of non-toxic, pharmacological modulators of cellular sphingolipid metabolism are available, including four FDA-approved compound, providing a possible starting point for studies addressing the question of whether cellular sphingolipid metabolism can be targeted in the context of host-directed antiviral therapeutics (95).

## MATERIALS AND METHODS

### Cells and viruses

Human hepatoma cells (Huh-7; Japanese Collection of Research Bioresources cell bank), human embryonal kidney cells (HEK-293T; ATCC CRL-1573), and human lung adenocarcinoma cells (A549; ATCC CCL-185) overexpressing the ACE2 receptor (Huh-7-ACE2, HEK-293T-ACE2, A549-ACE2; kindly provided by Friedemann Weber, Institute of Virology, Justus Liebig University Giessen, Germany), A549 overexpressing CD13 and TMPRSS2 (A549-CD13; kindly provided by Krzysztof Pyrć, Małopolska Centre of Biotechnology, Jagiellonian University, Kraków, Poland), and primary human lung fibroblasts (MRC-5 cells; ATCC CCL-171) were grown in Dulbecco’s modified Eagle’s medium (DMEM, Invitrogen) and supplemented with 10% fetal calf serum (FCS) and antibiotics (100 U/mL of penicillin, 100 µg/mL of streptomycin and 0.5 µg/mL puromycin).

HCoV-229E was obtained from the virus collection of the Institute of Medical Virology, Giessen, Germany. SARS-CoV-2 (isolate Munich 929) and MERS-CoV (EMC/2012) were kindly provided by Christian Drosten (Institute of Virology, Charité-Universitätsmedizin, Berlin, Germany).

### Inhibitors

The selective nSMase2 inhibitor PDDC (10 mM [44]) was purchased from ProbeChem Biochemicals Co Ltd. The nSMase inhibitor GW4869 (5 mM [42]) and 3-O-Sphingomyelin (10 mM [40]) were purchased from Sigma Aldrich and Enzo Life Science. The aSMase inhibitors ARC39 (1 mM [35]) and PCK310 (100 µM [38]) were kindly provided by Christoph Arenz (Institute of Chemistry, Humboldt University Berlin, Germany). The specific cPLA2α inhibitor Py-2 (20 mM [50]) was purchased from Merck Millipore. The inhibitor of nidoviral RO-formation K22 (20 mM [48]) was purchased from ProbeChem Biochemicals Co Ltd. Compounds were dissolved in DMSO or PBS and stored at −80°C.

### Cell viability assay

The cytotoxic effects of all the inhibitors used in this study were determined in a 96-well format by 3-(4,5-dimethyl-2-thiazolyl)-2,5-diphenyl-2H-tetrazolium bromide assay as described before (96). Briefly, cells were seeded in 96-well plates, grown until confluency, and then incubated with a cell culture medium containing the respective compound at the indicated concentrations. Following incubation for 24 h at 37°C, the culture medium was replaced with 200 µL DMEM containing 10% FBS and 175 µg/mL tetrazolium bromide (Sigma Aldrich). After 60–90 min of incubation at 37°C, the cells were fixed with 3.7% paraformaldehyde (PFA; Roth) for 30 min, and 200 µL isopropanol was added to each well. Formazan formation was measured by determining the absorbance at 490 nm using a spectrophotometer (BioTek). To determine cytotoxic concentration 50% (CC_50_) values, the obtained values were calculated in percentage with the respective DMSO control set as 100%. CC_50_ values were then calculated using non-linear regression analysis using GraphPad Prism 9.2 (GraphPad Software).

### Antiviral assay

To determine the effect of the indicated inhibitors, cell cultures were infected with the respective virus at an MOI of 0.1 pfu/cell in FCS-free DMEM at 33°C or 37 °C. After 1 hpi, the inoculum was removed, and the cells were incubated with FCS-free DMEM containing the different inhibitor concentrations. Supernatants were collected at 24 hpi, and virus titers were analyzed by plaque assay.

For virus titration using plaque assays, cells were seeded in 24-well plates. Following incubation for 24 h, the cells were inoculated with 10-fold virus dilutions in FCS-free DMEM. At 1 hpi, the virus inoculum was replaced with an Avicel-containing medium (1× MEM [Gibco], 1.25% Avicel [FMC Biopolymer] containing 10% FBS and antibiotics). At 24 hpi, the plates were washed with PBS, fixed with 3.7% PFA in PBS, and the cell layer was stained with 0.15% crystal violet.

### Immunofluorescence

Huh-7-ACE2 cells were seeded on top of coverslips in a 24-well plate. After transfection or virus infections with HCoV-229E, SARS-CoV-2, or MERS-CoV with an MOI of 3 pfu/cell, cells were washed with PBS and fixed with PBS containing 3.7% PFA and 0.5% Triton-X100 (or 0.5% saponin in case of ceramide staining) for 24 h at 4°C. Cells were washed with PBS and incubated with the appropriate primary antibody (1:100) in PBS containing 3% bovine serum albumin (BSA) for 24 h at 4°C. For the determination of viral infection rates, a mouse anti-HCoV-229E nucleocapsid protein mAb (Batch 250609, Ingenasa), mouse anti-SARS-CoV nucleocapsid protein mAb (MA5-29981; Invitrogen), or mouse anti-MERS-CoV nucleocapsid protein mAb (40068-MM10; Sino Biological) was used. For visualization of viral ROs, the mouse anti-dsRNA mAb (J2; SCICONS English and Scientific Consulting Kft.) was used. For the visualization of tagged nsp3 and nsp4, mouse anti-HA mAb (26183, Invitrogen) and rabbit anti-V5 mAb (13202, Cell Signal Technology) were used. For Cer visualization, mouse anti-ceramide mAb (ALX-804-196-Z050, Enzo) was used. Next, cells were washed and incubated with the appropriate secondary antibody (1:500; Alexa Fluor 488 goat anti-mouse IgG [H + L] [A11001, Invitrogen], Alexa Fluor 594 goat anti-mouse IgG [H + L] [A11005, Invitrogen], Alexa Fluor 488 F[ab′]2-goat anti-rabbit IgG [H + L] [A11070, Invitrogen]) in PBS containing 3% BSA for 2 h at RT. Cell nuclei were stained using DAPI (1:1000, Sigma-Aldrich). Finally, coverslips were washed with PBS and mounted on glass slides using ProLong Gold Antifade Mountant (P36934, Thermo Fisher).

Data were processed using the Imaris 8.4 software package (Bitplane). Colocalization rates between dsRNA, HA-nsp3, and nsp4-V5 signals with signals of Cer, EQ-SM, and nSMase2 were calculated for the total area of representative images using automated threshold settings. In addition, the corresponding Manders correlation coefficient was calculated for the total area of representative images.

### Quantification of sphingolipids by liquid chromatography tandem-mass spectrometry

Huh-7-ACE2, A549-ACE2, A549-CD13 or MRC-5 cells (1 × 10^6^ cells/well) were infected with the indicated virus at an MOI of 3 pfu/cell. At the indicated time points, cells were harvested, resuspended in 500 µL methanol, and subjected to sphingolipid extraction as described previously (97). To this end, 1 mL methanol/chloroform (1:1 [vol/vol]) was added that contained the internal standards d_7_-dihydrosphingosine (d_7_-dhSph), d_7_-sphingosine (d_7_-Sph), d_7_-sphingosine 1-phosphate (d_7_-S1P), 17:0 ceramide (d18:1/17:0), d_31_-16:0 sphingomyelin (d18:1/16:0-d_31_), 17:0 glucosyl(β) ceramide (d18:1/17:0), and 17:0 lactosyl(β) ceramide (d18:1/17:0; Avanti Polar Lipids). Final extracts were subjected to liquid chromatography tandem-mass spectrometry sphingolipid quantification by applying the multiple reaction monitoring approach. Chromatographic separation was achieved on a 1290 Infinity II HPLC (Agilent Technologies) equipped with a Poroshell 120 EC-C8 column (3.0 × 150 mm, 2.7 µm; Agilent Technologies) guarded by a pre-column (3.0 × 5 mm, 2.7 µm) of identical material. MS/MS analyses were carried out using a 6495C triple-quadrupole mass spectrometer (Agilent Technologies) operating in the positive electrospray ionization mode (ESI+). Chromatographic conditions and settings of the ESI source and MS/MS detector have been published elsewhere (97). The mass transitions used for the analysis of sphingolipid subspecies are given in Table S2. Peak areas of Cer, dhCer, SM, dihydrosphingomyelin (dhSM), hexosylceramide (HexCer), and lactosylceramide (LacCer) subspecies, as determined with MassHunter software (version 10.1, Agilent Technologies), were normalized to those of their internal standards followed by quantification via external calibration. DhSph, Sph, and S1P were directly quantified via their deuterated internal standards.

### siRNA-based knockdowns

For the genetic knockdown, Huh-7-ACE2 cells were transfected with 100 nM (50 nM in case of CD13) endonuclease-prepared siRNAs (aSMase/SMPD1 (EHU122691, MISSION; Sigma Aldrich), nSMase1/SMPD2 (EHU120741, MISSION; Sigma Aldrich), nSMase2/SMPD3 (EHU024631, MISSION; Sigma Aldrich), nSMase3/SMPD4 (EHU116241, MISSION; Sigma Aldrich), CD13/ANPEP (EHU037211, MISSION; Sigma Aldrich), ACE2 (EHU033081, MISSION; Sigma Aldrich), and DPP4 (EHU058011, MISSION; Sigma Aldrich) using Lipofectamine RNAiMAX reagent (Invitrogen). An equivalent amount of MISSION small-interfering RNA universal negative control 1 (SIC002, MISSION; Sigma Aldrich) was used as a negative control. Knockdown efficiency was validated via RT-qPCR (see “Quantitative real-time PCR”). At 48 h post-transfection, cells were infected with the indicated CoV (MOI = 0.1 pfu/cell). At 12 hpi, transfected and infected cells were fixed with 3.7% PFA and 0.5% Triton-X100 and stained with antibodies against the N protein of the respective CoV (see immunofluorescence). The percentage of infected cells was analyzed using the ImageXpress Pico automated cell imaging system (Molecular Devices) and calculated in relation to untreated control.

### Quantitative real-time PCR

Total RNA from Huh-7-ACE2 cells was isolated at 24 and 48 h post-transfection using the RNeasy Midi Kit (Qiagen), according to the manufacturer’s protocol. For the detection of specific mRNAs, the following primer pairs were used: aSMase (fwd 5′-GCTGGCTCTATGAAGCGATGGC-3′, rev 3′-AGAGCCAGAAGTTCTCACGGGA-5′), nSMase1 (fwd 5′-CAGGAGCTTACCCAGCACAT-3′, rev 3′-GTTGAGCACCATGCCA CTTA-5′), nSMase2 (fwd 5′-CGCATTCAAGGGCAATTACATTTT-3′, rev 3′-AAGTGA GGGTTGGTTTTGTGATT-5′), nSMase3 (fwd 5′-TGGAGATGTGGCTGAGCTACCT-3′, rev 3′-CAGCAGGTTCTCCTGGACAAAG-5′), CD13 (fwd 5′-GTAATACGACTCAC TATAGGGCCAGGG-3′, rev 3′-AATTAACCCTCACTAAAGGGCCACCAG-5′), ACE2 (fwd 5′-TGGGACTCTGCCATTTACTTAC-3′, rev 3′-CCCAACTATCTCTCGCTTCA TC-5′), DPP4 (fwd 5′-GGCACCTGGGAAGTCATCGGGA-3′, rev 3′-AGAGGGG CAGACCAGGACCG-5′), and GAPDH (fwd 5′-CTGCTCCTCCTGTTCGACAGT-3′, rev 3′-CCGTTGACTCCGACCTTCAC-5′). The 4× CAPITAL 1-Step qRT-PCR Green Master Mix (Biotechrabbit) was used for the detection of aSMase, nSMase1, nSMase3, CD13, ACE2, and DPP4. The Luna Universal One-Step RT-qPCR Kit (NEB) was used for the detection of nSMase2. The specificity of the reaction for each primer pair was confirmed by melting curve analysis and agarose gel electrophoresis. Mean Ct values from technical duplicates of each sample were normalized to the housekeeping control GAPDH. Fold change gene expression compared to the negative control was then calculated using the delta delta *C*_*t*_ method.

### Plasmid construction and transfection

For the transient expression of the nsp3-4-fusionprotein, a codon-optimized sequence of HCoV-229E nsp3-4 was synthesized in a pcDNA3.1 vector (BioCat). To allow detection of the proteins, the coding sequences for an N-terminal HA-tag and a C-terminal V5-tag were added. The other constructs encoding for the single proteins and non-cleavable polyprotein were generated by PCR. Details on the HCoV-229E ORF1a-encoded amino acid sequences included in the different expression constructs are given in Table S1 in the supplemental material. The plasmid DNA construct encoding for PC4-ss-FM4-EQ-SM-oxGFP was kindly provided by Christopher Burd (Department of Cell Biology, Yale University, New Haven, CT). The constructs encoding nSMase2-eGFP (pcDNA3.1-nSMase2-eGFP), the catalytically inactive mutant nSMase2(H639A)-eGFP (pcDNA3.1-nSMase2[H639A]-eGFP), and pcDNA3.1-eGFP were kindly provided by Vera Kozjak-Pavlovic (Chair of Microbiology, Julius Maximilian University Würzburg, Germany). The empty vector control pcDNA3.1 was purchased from Addgene.

For transfection of the plasmids for immunofluorescence analysis, Huh-7-ACE2 cells were seeded on coverslips and transfected with 0.75 µg plasmid DNA and 2 µL Lipofectamine 2000 (Invitrogen, Thermo Fisher), mixed in serum-reduced Opti-MEM (Gibco, Thermo Fisher). For western blot analysis, HEK-293T-ACE2 cells (1 × 10^6^ cells) were transfected with 2.5 µg DNA and 10 µL Lipofectamine 2000, mixed in serum-reduced Opti-MEM.

### Western blotting

HEK-293T-ACE2 cells were transfected as described above. At 24 hpi, the cells were lysed in membrane-solubilizing lysis buffer (50 mM Tris-HCl, 150 mM NaCl, 1 mM EDTA, 0.5% dodecylmaltoside [ThermoFisher Scientific], and 5% glycerol). Proteins were separated by SDS-PAGE in an 8 and 12% hybrid gel and transferred onto a 0.45 μm pore-size nitrocellulose membrane (GE Healthcare). Membranes were incubated overnight at 4°C with mouse anti-HA mAb (26183, Invitrogen) or rabbit anti-V5 mAb (13202, Cell Signal Technology) diluted in PBS containing 3% BSA. After extensive washing with PBS, the membrane was incubated with goat anti-rabbit IRDye 800CW (1:10,000, 926-32211, LI-COR) and goat anti-mouse IRDye 680CW (1:10,000, 926-68070, LI-COR) polyclonal antibodies for 2 h at room temperature. After another extensive washing with PBS, the immunostained proteins were visualized using a LI-COR Odyssey imaging system and software.

### Electron microscopy

For transmission electron microscopy (TEM) analysis of artificial ROs, Huh-7-ACE2 cells (2 × 10^6^ cells) were transfected with 3 µg plasmid DNA and 9 µg PEI STAR (Tocris Bioscience). Cells were fixed at 24 h post-transfection with 1.5% PFA and 1.5% glutaraldehyde (Sigma Aldrich) in 0.15 M HEPES, scraped, and centrifuged at 1,000 × *g* for 10 min. Next, the cell pellet was washed and post-fixed in a buffer containing 1% osmium tetroxide (Merck). After washing in distilled water, the samples were incubated overnight in 2% aqueous uranyl acetate (Merck) at 4°C, dehydrated in ethanol, and embedded in AGAR 100 (Agar Scientific Ltd, Essex, UK). Ultrathin sections were mounted on grids and analyzed using a transmission electron microscope (LEO EM 906, LEO Elektronenmikroskopie GmbH-Zeiss, Oberkochen, Germany) equipped with a slow-scan 2 K CCD camera (TRS, Tröndle, Moorenweis, Germany).

### Data analysis

Statistical parameters (means, *t*-tests, standard variations, and non-linear fittings for CC_50_ values) were calculated using GraphPad Prism 9.2 or Microsoft Excel 2016.

## SUPPLEMENTAL MATERIAL

Fig. S1 mbio.00084-25-s0001.tif Antiviral activities of a/nSMase inhibition in CoVs replication.

Fig. S2 mbio.00084-25-s0002.tif Effect of cPLA2a inhibition using Py-2 on RO formation.

Fig. S3 mbio.00084-25-s0003.tif Colocalization of ROs with pcDNA3.1-eGFP in CoV-infected cells.

Fig. S4 mbio.00084-25-s0004.tif Colocalization of ROs with enzymatically inactive nSMase_GFP (H639A) in CoV-infected cells.

Table S1 mbio.00084-25-s0005.tif Details of HCoV-229E expression constructs.

Table S2 mbio.00084-25-s0006.tif LC-MS/MS parameter for sphingolipid quantification.

ASM does not own the copyrights to Supplemental Material that may be linked to, or accessed through, an article. The authors have granted ASM a non-exclusive, world-wide license to publish the Supplemental Material files. Please contact the corresponding author directly for reuse.
