# Supplementary figures and images for: Targeting sphingolipid metabolism: inhibition of neutral sphingomyelinase 2 impairs coronaviral replication organelle formation
Source: mBio. 2025 Aug 14;16(9):e00084-25. doi: 10.1128/mbio.00084-25 (PMC12421859; doi:10.1128/mbio.00084-25)

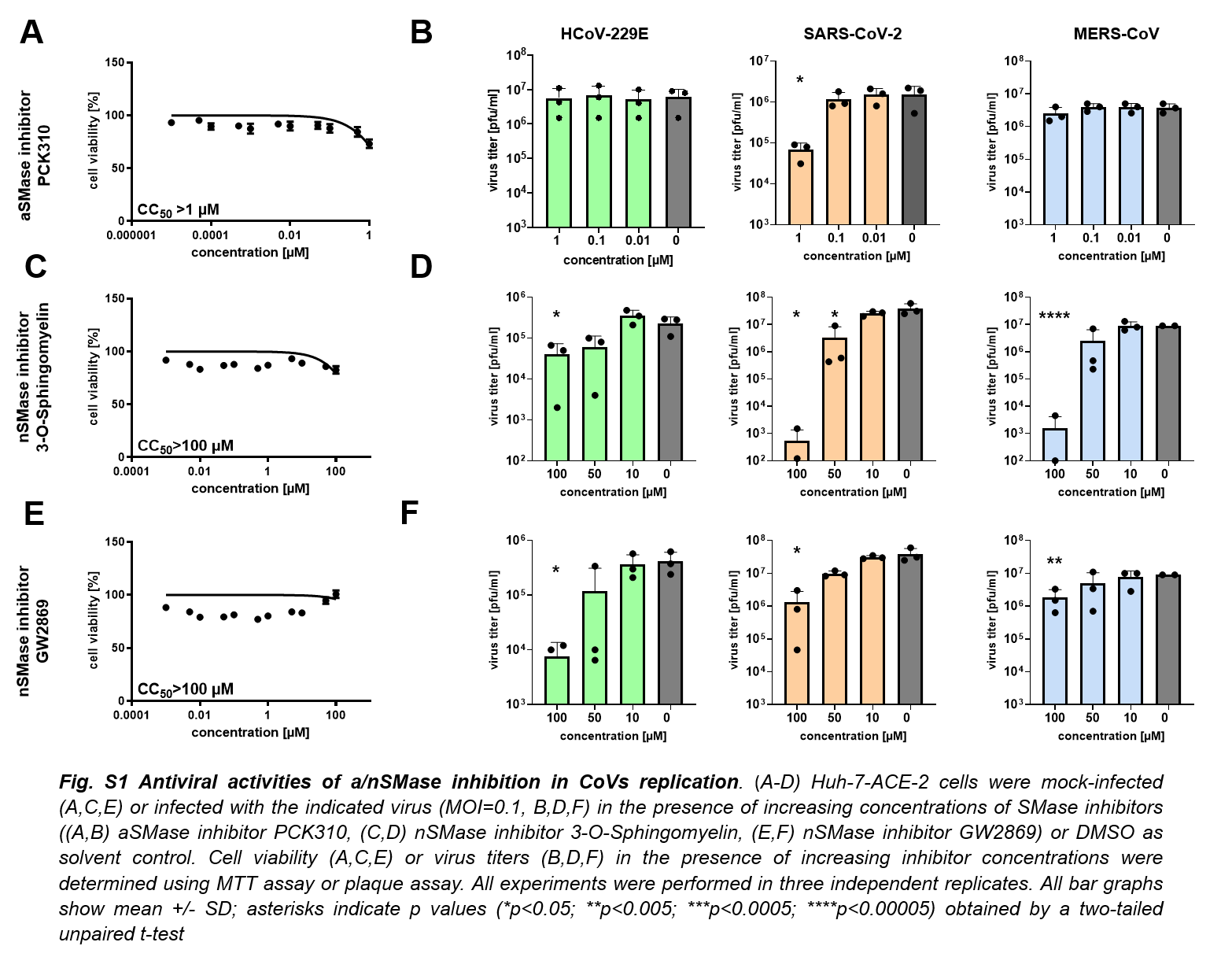

Supplement: Fig. S1 — Antiviral activities of a/nSMase inhibition in CoVs replication. [file mbio.00084-25-s0001.tif]

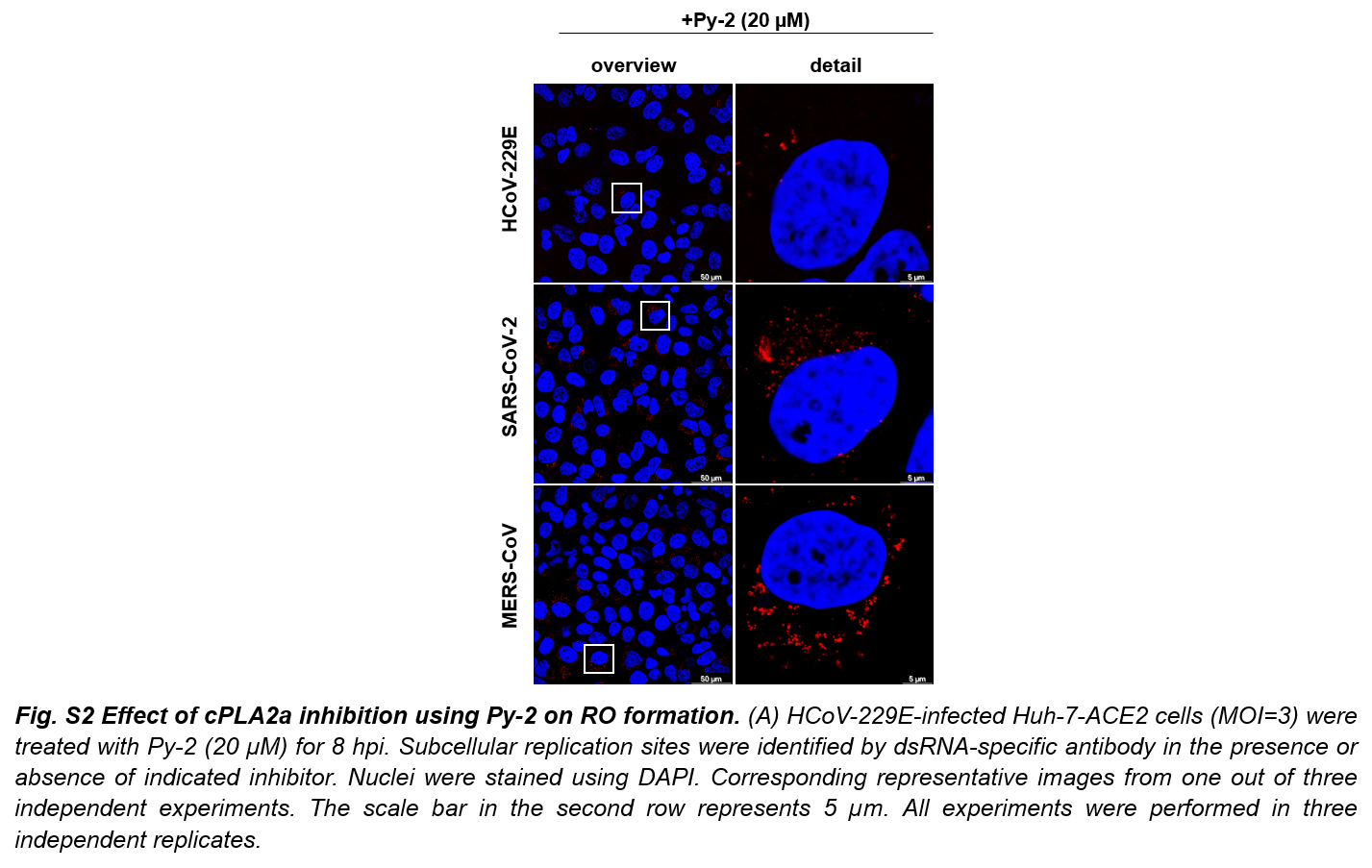

Supplement: Fig. S2 — Effect of cPLA2a inhibition using Py-2 on RO formation. [file mbio.00084-25-s0002.tif]

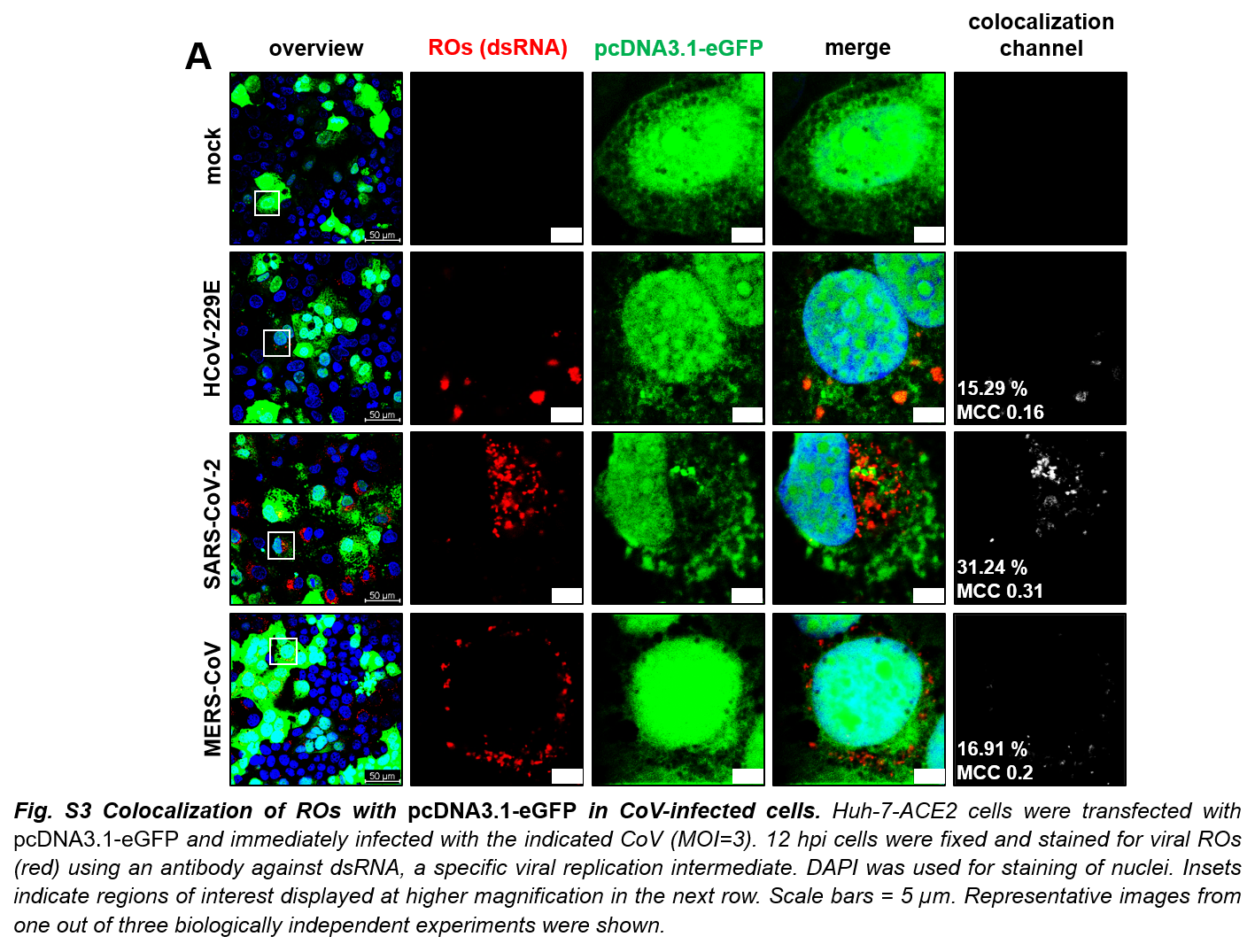

Supplement: Fig. S3 — Colocalization of ROs with pcDNA3.1-eGFP in CoV-infected cells. [file mbio.00084-25-s0003.tif]

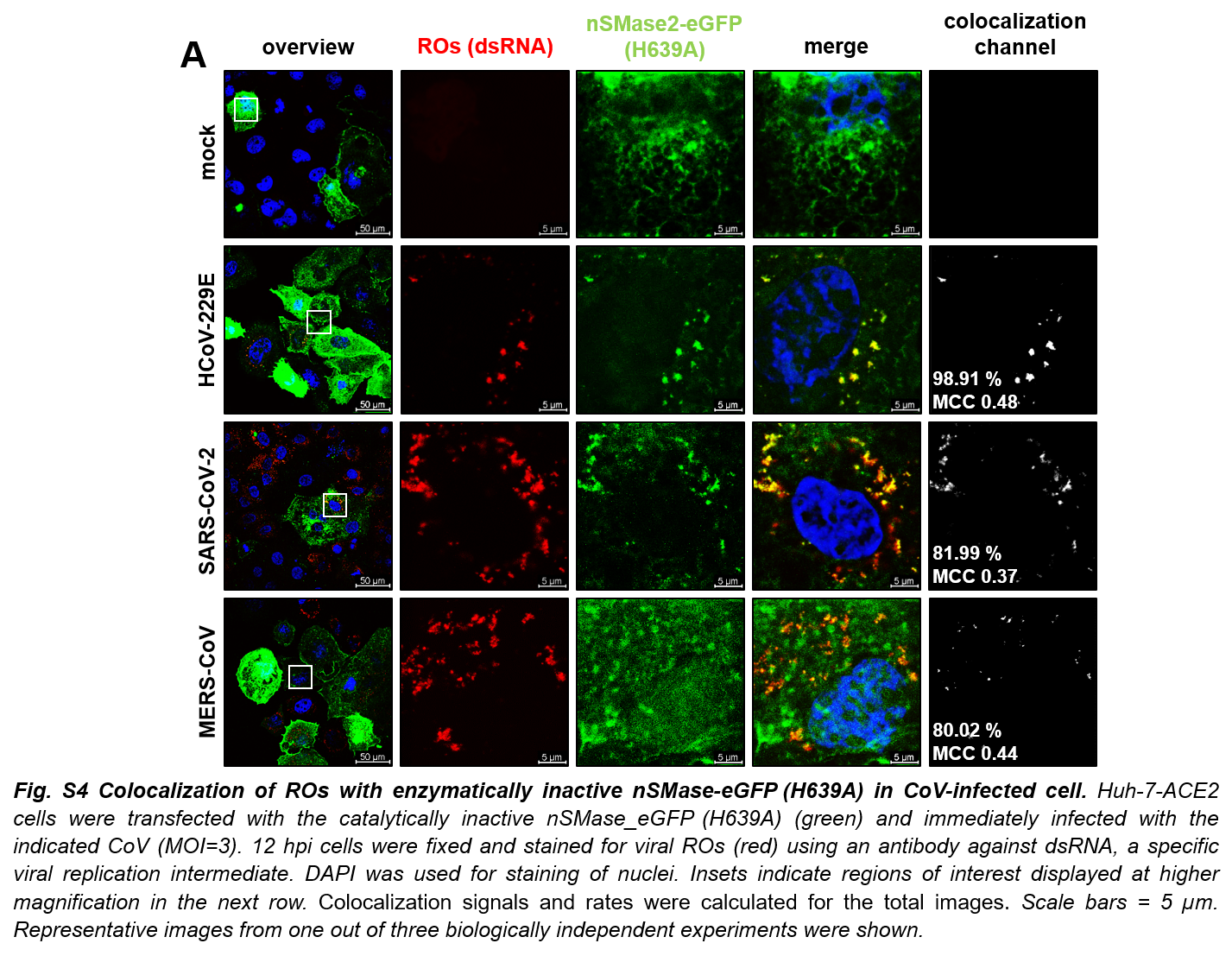

Supplement: Fig. S4 — Colocalization of ROs with enzymatically inactive nSMase_GFP (H639A) in CoV-infected cells. [file mbio.00084-25-s0004.tif]

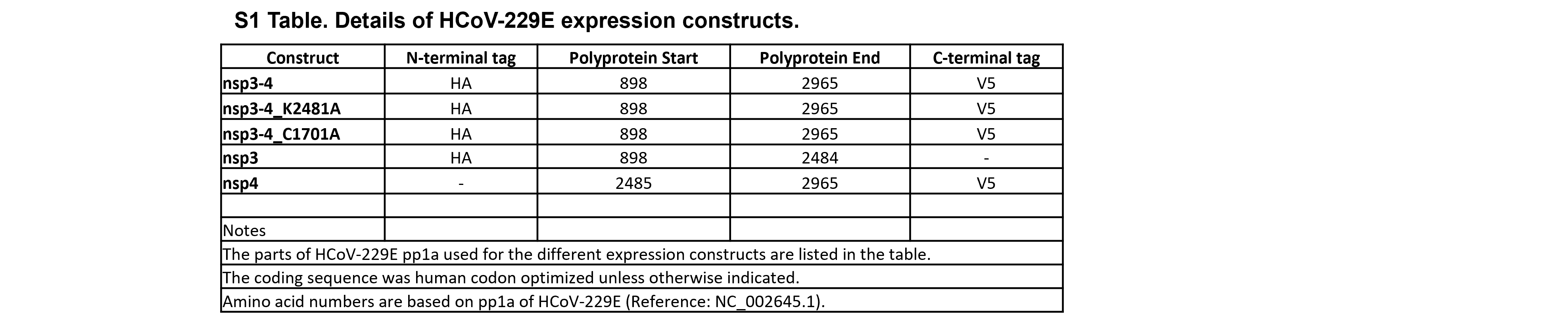

Supplement: Table S1 — Details of HCoV-229E expression constructs. [file mbio.00084-25-s0005.tif]

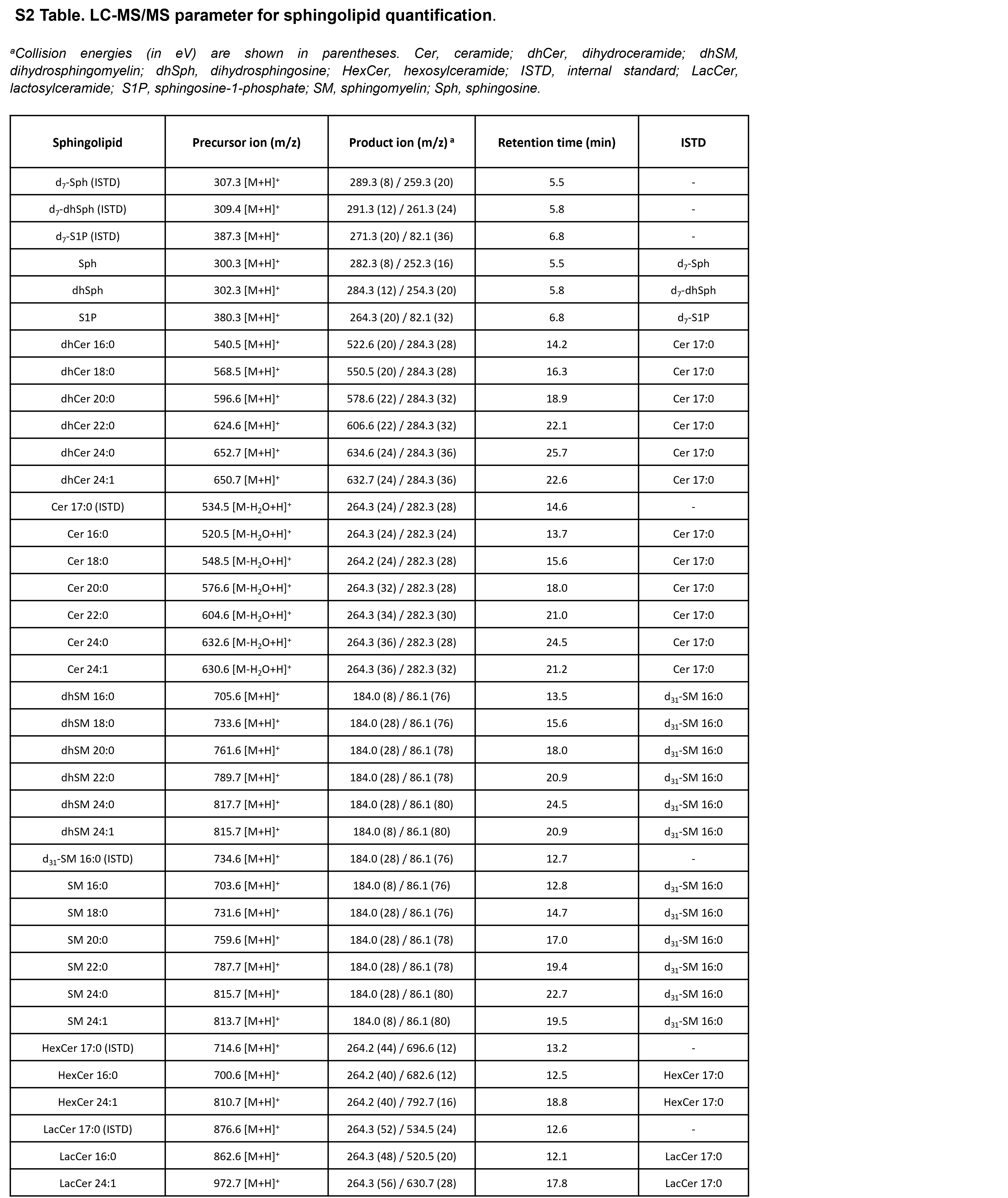

Supplement: Table S2 — LC-MS/MS parameter for sphingolipid quantification. [file mbio.00084-25-s0006.tif]
